# Preoperative risk stratification for postoperative inflammatory–nutritional deterioration after colorectal cancer surgery: an interpretable machine learning study

**DOI:** 10.3389/fmed.2026.1938414

**Published:** 2026-09-29

**Authors:** Yinchao Zhang, Yingkun Hu, Xinke Cao, Xinquan Zan, Zhiqiang Tian, Wei Shen

**Affiliations:** The Affiliated Wuxi People’s Hospital of Nanjing Medical University, Wuxi People’s Hospital, Wuxi Medical Center, Nanjing Medical University, Wuxi, Jiangsu, China

**Keywords:** colorectal cancer, machine learning, modified Glasgow prognostic score, postoperative inflammation, risk prediction, SHAP

## Abstract

**Background:**

Postoperative inflammatory–nutritional deterioration may adversely affect recovery after colorectal cancer surgery. This study aimed to develop and internally validate an interpretable machine learning model for preoperative prediction of postoperative modified Glasgow Prognostic Score (mGPS) = 2.

**Methods:**

This single-centre retrospective study included consecutive patients who underwent elective radical resection for colorectal cancer between 2020 and 2025. The cohort was divided into training and internal validation sets using stratified sampling. Recursive feature elimination identified eight predictors: age, prealbumin, albumin, cholinesterase, lymphocyte percentage, platelet count, fibrinogen and serum calcium. Eight conventional machine learning algorithms, TabICLv2 and SuperLearner ensembles were evaluated using discrimination, calibration and decision curve analyses. SHapley Additive exPlanations were used for model interpretation.

**Results:**

Among 1196 patients, 507 (42.4%) developed postoperative mGPS=2. In the internal validation cohort, TabICLv2 achieved an area under the receiver operating characteristic curve of 0.753 (95% confidence interval, 0.701–0.804) and a Brier score of 0.197. SuperLearner-W and SuperLearner-SR showed comparable discrimination, with areas under the curve of 0.752 and 0.751, respectively. Lymphocyte percentage, platelet count and fibrinogen were the leading contributors to model predictions. Learning-curve analysis revealed suboptimal convergence behaviour, with relatively flat cross-validation trajectories and persistent separation from training performance across increasing training fractions. A web-based research prototype was developed to provide individualised risk estimates, patient-level feature attribution and an applicability-domain assessment indicating the level of support from the model-development data.

**Conclusion:**

An interpretable model based on routinely available preoperative variables provided moderate discrimination for postoperative inflammatory–nutritional deterioration after colorectal cancer surgery. The suboptimal learning-curve convergence indicates that the model is not suitable for clinical deployment without further development and independent external validation.

## 1 Introduction

Colorectal cancer (CRC) remains one of the most prevalent gastrointestinal malignancies worldwide, and radical surgical resection continues to be the primary curative treatment for resectable disease. However, major surgical trauma can trigger a pronounced systemic inflammatory response syndrome (SIRS) (1). Regardless of whether the primary tumour is located in the colon or rectum, severe postoperative systemic inflammation and subsequent nutritional deterioration may adversely affect the prognosis of patients with CRC (2). In clinical practice, the modified Glasgow Prognostic Score (mGPS), which integrates C-reactive protein (CRP) and albumin (ALB) levels, is a widely used biomarker reflecting systemic inflammation and nutritional impairment. An mGPS of 2 indicates a high-risk inflammatory–nutritional status characterised by elevated CRP and hypoalbuminaemia (3). Elevated preoperative and postoperative mGPS levels have been associated with poorer overall and disease-free survival, as well as short-term adverse outcomes, including postoperative infection, anastomotic leakage, and surgical site infection (SSI) (4, 5). This issue may be particularly important in elderly or frail patients with CRC, in whom baseline inflammation and malnutrition may further increase the risk of severe postoperative complications (6, 7).

Beyond mGPS, peripheral blood-based indices of immune, inflammatory and coagulation status have been extensively evaluated for risk stratification in patients with CRC. Metrics such as the systemic immune-inflammation index (SII), neutrophil-to-lymphocyte ratio (NLR), platelet-to-lymphocyte ratio (PLR), fibrinogen (Fbg) and neutrophil-platelet score (NPS) have been widely investigated (8–11). These biomarkers may provide complementary value in predicting postoperative outcomes in CRC, and preoperative hypercoagulability and elevated inflammatory indices have been reported as risk factors for poor survival and adverse clinical outcomes (12–14). However, single conventional markers may be insufficient for predicting postoperative mGPS = 2, which represents a multidimensional high-risk status involving inflammation, nutritional depletion, immune dysfunction and coagulation activation. The nonlinear interactions among these biological processes limit the ability of traditional unidimensional markers to support accurate individualised risk prediction.

In recent years, machine learning (ML) approaches have shown increasing promise in perioperative risk prediction for patients undergoing colorectal cancer surgery, including the prediction of surgical site infection, acute kidney injury, long-term survival and treatment-related outcomes (15–18). Recent studies have further extended machine-learning-based risk assessment beyond baseline patient characteristics by incorporating procedural and perioperative determinants. In a 2025 multicentre study of laparoscopic right hemicolectomy for colon cancer, Anania et al. developed deep-learning models for postoperative complications and identified surgical and perioperative factors, including intraoperative bleeding, blood transfusion, anastomotic technique and adherence to fast-track recovery pathways, as relevant predictors (19). In the same year, Violante et al. demonstrated that a Super Learner framework improved prediction of multiple postoperative outcomes in colorectal surgery compared with conventional logistic regression and XGBoost approaches (20). More recently, Elengickal et al. showed that surgical approach and operative duration may interact to define distinct procedural risk phenotypes, with prolonged open procedures carrying particularly high postoperative complication risk (21). A 2026 multicentre machine-learning study of emergency colon cancer surgery likewise identified surgical approach together with intraoperative physiological disturbances and inflammatory markers as important determinants of postoperative sepsis (22). Collectively, these studies indicate that postoperative risk reflects not only preoperative patient susceptibility but also the magnitude and characteristics of the subsequent procedural stress. However, preoperative identification of patients susceptible to marked postoperative inflammatory–nutritional deterioration remains insufficiently studied. Such risk stratification may support structured nutritional assessment, closer perioperative monitoring and individualised supportive care. Therefore, this study aimed to develop and internally validate an interpretable model for predicting postoperative mGPS = 2 using routinely available preoperative variables. Conventional machine learning algorithms, the TabICLv2 tabular foundation model and SuperLearner ensembles were evaluated using discrimination, calibration and decision curve analyses. SHapley Additive exPlanations (SHAP) were used to examine global and patient-level feature attribution, and a web-based calculator was developed as a research prototype for individualised perioperative risk estimation.

## 2 Materials and methods

### 2.1 Study design and ethical approval

This single-centre, retrospective cohort study included consecutive patients with colorectal cancer (CRC) who underwent elective radical resection at The Affiliated Wuxi People's Hospital of Nanjing Medical University, Wuxi People's Hospital, Wuxi Medical Center, Nanjing Medical University, between January 2020 and December 2025. Initial screening of the institutional electronic medical record (EMR) system identified 1600 potentially eligible patients. After applying the predefined inclusion and exclusion criteria, a final cohort of 1,196 patients was included for model development and internal validation.

The study protocol was conducted in accordance with the Declaration of Helsinki and was approved by the Ethics Review Committee of Wuxi Medical Center, Nanjing Medical University (Approval No. KY26124). Given the retrospective design of the study and the use of de-identified clinical data, the requirement for written informed consent was waived by the institutional review board.

This manuscript was prepared with reference to the TRIPOD + AI reporting principles for clinical prediction model studies using regression or machine learning methods (23).

### 2.2 Study population

Patients were included if they met all of the following criteria:

1. Age ≥ 18 years.
2. Primary colorectal cancer confirmed by preoperative colonoscopic biopsy or postoperative pathology.
3. Initial elective radical resection for colorectal cancer, including either laparoscopic or open surgery.

Patients were excluded if they met any of the following criteria:

1. Non-elective surgery, including emergency surgery for tumour-related complications such as acute bowel obstruction, intestinal perforation or severe gastrointestinal haemorrhage.
2. Confirmed preoperative mGPS = 2, defined as preoperative CRP > 10 mg/L together with albumin (ALB) < 35 g/L. Eligibility was determined using the original observed preoperative CRP and ALB measurements before data splitting and missing-value imputation. Imputed values were not used to determine this exclusion criterion.
3. Severe major organ dysfunction, including end-stage renal disease requiring maintenance dialysis, decompensated liver cirrhosis (Child–Pugh class B or C), acute severe hepatitis, haematological malignancy or active long-term immunosuppressive therapy.
4. Indeterminate endpoints, defined as cases lacking the critical postoperative laboratory data required to determine postoperative mGPS = 2. When one component of the composite endpoint was missing but the available paired component definitively ruled out the joint positive condition, the patient was classified as not having postoperative mGPS = 2 and retained in the cohort.

The detailed patient selection process and model development workflow are shown in Figure 1.

**Figure 1:**
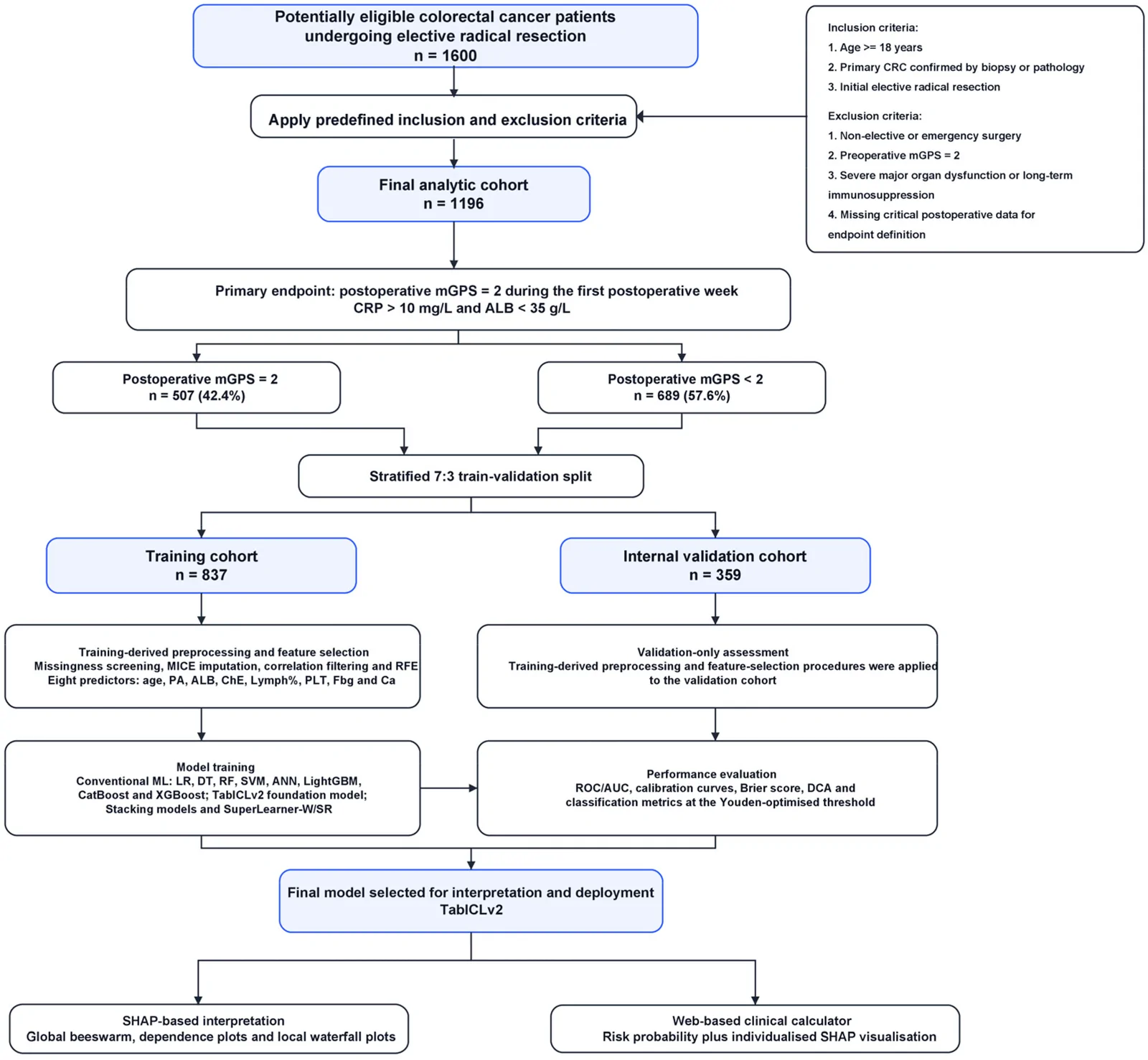
Flowchart of the study population selection process and model development workflow. CRC, colorectal cancer; mGPS, modified Glasgow Prognostic Score; CRP, C-reactive protein; ALB, albumin; MICE, Multiple Imputation by Chained Equations; RFE, recursive feature elimination; PA, prealbumin; ChE, cholinesterase; Lymph%, lymphocyte percentage; PLT, platelet count; Fbg, fibrinogen; Ca, serum calcium; DCA, decision curve analysis; SHAP, SHapley Additive exPlanations.

### 2.3 Sample size considerations

No formal *a priori* sample size calculation was performed because this was a retrospective cohort study based on all eligible patients available in the institutional EMR system during the study period. The final cohort included 1,196 patients, of whom 507 developed postoperative mGPS = 2. The final model incorporated eight predictors. Because model development was performed within the training cohort, the full-cohort event count is reported descriptively rather than used as a formal events-per-predictor justification.

### 2.4 Data extraction and outcome definition

Clinical baseline data were retrieved from the institutional Yidu Cloud EMR system, covering assessments performed within one week before surgery. Extracted variables included patient demographics, vital signs, comorbidity history and laboratory metrics, including complete blood counts, biochemical panels, coagulation profiles and circulating tumour markers (Table 1). Anatomical tumour location was also collected as a clinically relevant covariate. To preserve information and avoid arbitrary categorisation, continuous laboratory parameters were retained as numerical variables.

**Table 1:** Baseline clinical and laboratory characteristics of the study population according to postoperative mGPS status.

Characteristics	mGPS = 2 (n=507)	mGPS < 2 (n=689)	P value	SMD
Sex, n (%)			0.885	0.008
Female	201 (39.6%)	276 (40.1%)		
Male	306 (60.4%)	413 (59.9%)		
Diagnosis, n (%)			0.004	0.169
Colon cancer	305 (60.2%)	357 (51.8%)		
Rectal cancer	202 (39.8%)	332 (48.2%)		
Hypertension, n (%)			0.221	0.072
No	289 (57.0%)	417 (60.5%)		
Yes	218 (43.0%)	272 (39.5%)		
Diabetes, n (%)			0.362	0.053
No	433 (85.4%)	601 (87.2%)		
Yes	74 (14.6%)	88 (12.8%)		
Age, median (IQR)	70.83 (62.25, 77.41)	64.58 (57.25, 71.33)	<0.001	0.500
Temperature, median (IQR)	36.6 (36.5, 36.8)	36.6 (36.5, 36.8)	0.405	0.053
Pulse, median (IQR)	78 (74.5, 86)	78 (74, 83)	0.024	0.158
Albumin, median (IQR)	38.5 (35.5, 40.6)	39.8 (38.8, 43.1)	<0.001	0.634
Prealbumin, median (IQR)	196.8 (150.4, 234.6)	223.7 (199, 261.1)	<0.001	0.629
Cholinesterase, median (IQR)	6,405 (5,264, 7,358)	7,048 (6,280, 8,059)	<0.001	0.542
Lymphocyte percentage, median (IQR)	23.7 (17.85, 31.05)	25.7 (21.9, 31.4)	<0.001	0.249
Platelet count, median (IQR)	237 (190, 301)	247 (204, 281)	0.959	0.072
Fibrinogen, median (IQR)	3.21 (2.78, 3.75)	3.08 (2.71, 3.34)	<0.001	0.365
Serum calcium, median (IQR)	2.23 (2.145, 2.3)	2.27 (2.22, 2.36)	<0.001	0.528
C-reactive protein, median (IQR)	3.1 (1, 9.6)	2 (0.6, 6.1)	<0.001	0.430
D-dimer, median (IQR)	192 (110.5, 429.5)	136 (83, 257)	<0.001	0.368
CEA, median (IQR)	4.55 (2.14, 15.88)	3.55 (1.87, 14.01)	0.005	0.161
CA125, median (IQR)	10.6 (7, 19.05)	9.3 (6.2, 15.3)	<0.001	0.096

mGPS, modified Glasgow Prognostic Score; IQR, interquartile range; CRP, C-reactive protein; CEA, carcinoembryonic antigen; CA125, carbohydrate antigen 125; SMD, standardised mean difference. Continuous variables are presented as median (IQR), and categorical variables are presented as n (%). *P* values were calculated using the Mann–Whitney *U* test for continuous variables and Pearson's chi-square test, Yates' continuity correction or Fisher's exact test for categorical variables, as appropriate. SMD values are presented as absolute standardised differences, and an absolute SMD greater than 0.10 was considered indicative of a meaningful between-group difference. The complete baseline comparison is provided in Supplementary Table S1.

The primary endpoint was postoperative mGPS = 2 during the first postoperative week. For each postoperative measurement occasion with paired serum C-reactive protein (CRP) and albumin (ALB) results, mGPS = 2 was defined as CRP > 10 mg/L together with ALB < 35 g/L. A patient was classified as having postoperative mGPS = 2 if this combination was observed on at least one paired measurement occasion during the first postoperative week. This endpoint was interpreted as postoperative inflammatory–nutritional deterioration.

### 2.5 Statistical analysis and pre-processing

Descriptive statistics and data pre-processing were performed using R version 4.2.3 and Python version 3.10.11. Model development, hyperparameter optimisation, model evaluation and visualisation were performed using Python packages including pandas version 2.3.3, NumPy version 2.0.2, scikit-learn version 1.6.1, LightGBM version 4.6.0, CatBoost version 1.2.10, XGBoost version 2.1.4, Optuna version 4.7.0, SHAP version 0.49.1, tabicl version 2.0.2, statsmodels version 0.14.6, matplotlib version 3.9.4, seaborn version 0.13.2 and Streamlit version 1.50.0.

Eligibility criteria, including exclusion of patients with confirmed preoperative mGPS = 2, were applied using the original observed data before any missing-value imputation. Variables with missingness exceeding 30% were excluded before model development. After stratified data splitting, missing values in the retained candidate predictor variables were imputed using Multiple Imputation by Chained Equations (MICE). The imputation models and all subsequent preprocessing, feature selection and hyperparameter optimisation procedures were fitted exclusively within the training cohort and subsequently applied to the internal validation cohort to minimise information leakage. A fixed random seed was used for data splitting, model training, cross-validation and bootstrap resampling where applicable.

The Shapiro–Wilk test was used to assess the normality of continuous variables for sample sizes ≤5000. Normally distributed continuous variables were expressed as the mean ± standard deviation (SD), and between-group comparisons were performed using Student's *t* test for equal variances or Welch's *t* test for unequal variances, as determined by Levene's test. Non-normally distributed continuous variables were presented as medians with interquartile ranges (IQR) and compared using the Mann–Whitney *U* test. Categorical variables were summarised as frequencies and percentages. Comparisons were conducted using Pearson's chi-square test, Yates' continuity correction or Fisher's exact test, according to sample size and expected frequencies.

To complement statistical significance testing, standardised mean differences (SMDs) were calculated to quantify the magnitude of between-group differences in baseline characteristics. For continuous variables, SMDs were calculated as the difference in group means divided by the pooled standard deviation. For binary or categorical variables, SMDs were calculated based on differences in proportions; for multicategorical variables, the maximum absolute level-specific SMD was reported. SMD values were presented as absolute standardised differences, and an absolute SMD greater than 0.10 was considered indicative of a meaningful between-group difference. All statistical tests were two-sided, and a *P* value < 0.05 was considered statistically significant.

### 2.6 Feature selection, ensemble modelling and interpretability

To reduce the influence of multicollinearity, pairwise Pearson correlation analysis was performed for continuous baseline variables, with Benjamini–Hochberg false discovery rate (FDR) correction applied to correlation *P* values. Highly correlated redundant features, defined as Pearson |r|>0.70 with FDR-adjusted P<0.05, were removed based on clinical relevance and data completeness.

After predefined exclusions and initial missingness screening, the dataset was partitioned into a training cohort (70%) and an internal validation cohort (30%) using stratified sampling to preserve the distribution of the target outcome. Within the training cohort, missing-value imputation and feature filtering were performed before feature selection. Random-forest-based recursive feature elimination was subsequently conducted using five-fold cross-validation. Candidate feature subsets were evaluated according to the mean cross-validated AUC, and the eight-variable subset was retained for subsequent model development.

All imputation parameters, feature filtering procedures, feature selection steps and hyperparameter optimisation processes were derived exclusively from the training cohort and subsequently applied to the internal validation cohort to minimise information leakage.

Following feature selection, eight conventional machine learning classifiers were constructed and trained: Logistic Regression (LR), Support Vector Machine (SVM), Decision Tree (DT), Random Forest (RF), Artificial Neural Network (ANN), LightGBM, CatBoost and XGBoost. Hyperparameter optimisation for these models was performed using the Optuna framework with five-fold cross-validation, targeting maximisation of the area under the receiver operating characteristic curve (AUC).

In parallel, this study evaluated TabICLv2, a recently developed tabular foundation model using an in-context learning framework for tabular prediction tasks (24). In this study, TabICLv2 was used as a candidate classifier and was evaluated in the same training-validation framework as the conventional machine learning models.

To further explore model fusion, two intermediate stacking ensembles were constructed: a classic stacking model integrating SVM and CatBoost, and a heterogeneous stacking model integrating TabICLv2 with XGBoost. Subsequently, a SuperLearner ensemble framework was developed using out-of-fold (OOF) predicted probabilities from base learners as meta-features. Two meta-learning strategies were used: SuperLearner-W, based on non-negative least squares (NNLS) to estimate model weights, and SuperLearner-SR, based on Ridge regression with L2 regularisation to reduce the influence of disproportionately high single-model weights and encourage predictive diversity.

To improve model interpretability, the SHapley Additive exPlanations (SHAP) framework was incorporated. SHAP was used to quantify global feature importance and examine how core laboratory indices contributed to postoperative mGPS = 2 through feature dependence plots.

### 2.7 Model evaluation

Discriminative performance was evaluated using receiver operating characteristic (ROC) curves and quantified by the area under the curve (AUC). The AUC was calculated using predicted probabilities, and the 95% confidence intervals were estimated using 1,000 bootstrap resamples, with the 2.5th and 97.5th percentiles used as the lower and upper confidence limits.

To complement ROC-based discrimination, precision–recall (PR) curves were additionally constructed in the internal validation cohort using continuous predicted probabilities. Average precision (AP) was calculated for each model to summarise performance across the precision–recall relationship. Because postoperative mGPS = 2 occurred in 152 of 359 patients (42.3%) in the internal validation cohort, an event prevalence of 0.423 was used as the no-skill reference for interpretation of the PR curves.

To further examine model learning behaviour, model-agnostic learning-curve analyses were performed for TabICLv2, Random Forest, CatBoost, XGBoost and LightGBM. Because these algorithms use fundamentally different training mechanisms and TabICLv2 does not undergo conventional task-specific iterative parameter optimisation, iteration-based learning curves were not directly comparable across models. Learning curves were therefore evaluated as a function of effective training-set size within the original training cohort. Using stratified five-fold cross-validation, 20%, 40%, 60%, 80% and 100% of each fold-specific training partition were used for model fitting, while the corresponding held-out fold was used for cross-validation assessment. Previously selected model specifications were retained without additional hyperparameter optimisation. Mean training and cross-validation AUCs and their standard deviations across the five folds were calculated at each training fraction. The independent internal validation cohort was not used in the learning-curve analysis.

The operating threshold was determined from out-of-fold predictions in the training cohort by maximising the Youden index, defined as sensitivity + specificity −1, and was subsequently applied unchanged to the internal validation cohort. Sensitivity, specificity, accuracy, precision, F1 score, Cohen's kappa, positive predictive value and negative predictive value were then calculated at the Youden-optimised threshold. Model calibration was assessed using calibration plots and quantified using the Brier score. Decision curve analysis (DCA) was used to estimate net benefit across a range of threshold probabilities and to characterise decision-analytic performance.

Because several leading models achieved closely comparable validation discrimination, selection of a representative model for downstream interpretation and prototype deployment was not based on AUC ranking alone. The selection considered discrimination, calibration, decision-curve performance, model architecture and practical deployment complexity. Accordingly, the selected model was intended to serve as a representative model for interpretation and implementation rather than to imply statistical superiority over competing models.

To further interpret the TabICLv2 model, global feature importance and the directional effects of individual predictors on postoperative mGPS = 2 were visualised using SHAP summary beeswarm plots. Local SHAP waterfall plots were generated to examine individualised risk attribution. Among the 150 interpreted patients in the validation cohort, the patient with the highest predicted probability and the patient with a predicted probability closest to the 10th percentile were selected as illustrative high- and low-risk patients, respectively.

#### 2.7.1 Mathematical definitions of key statistical and model evaluation metrics

For transparency and reproducibility, the main statistical and model evaluation metrics used in this study are defined below (Equations 1–14). The binary outcome for each patient was defined as

$$Y_{i}=\left\{ \begin{array}{ll} 1, & {\mathrm{CRP}}_{i}^{post}>10\mathrm{mg}/{L\;\mathrm{and}\;\mathrm{ALB}}_{i}^{post}<35g/L,\\ 0, & \mathrm{otherwise}, \end{array}\right.$$ (1)

where $Y_{i}=1$ indicated that the joint CRP and ALB criterion for postoperative mGPS = 2 was observed on at least one paired measurement occasion during the first postoperative week.

For continuous baseline variables, the standardised mean difference (SMD) was calculated as

$$\mathrm{SMD}=\left|\frac{{\bar{x}}_{1}-{\bar{x}}_{0}}{s_{p}}\right|,$$ (2)

where ${\bar{x}}_{1}$ and ${\bar{x}}_{0}$ are the group means in the postoperative mGPS = 2 and mGPS < 2 groups, respectively, and $s_{p}$ is the pooled standard deviation:

$$s_{p}=\sqrt{\frac{(n_{1}-1)s_{1}^{2}+(n_{0}-1)s_{0}^{2}}{n_{1}+n_{0}-2}}.$$ (3)

For binary or categorical variables, level-specific SMDs were calculated using group proportions:

$$\mathrm{SM}D_{l}=\left|\frac{\;p_{1l}-p_{0l}}{\sqrt{\{\;p_{1l}(1-p_{1l})+p_{0l}(1-p_{0l})\}/2}}\right|,$$ (4)

where $p_{1l}$ and $p_{0l}$ denote the proportions of category level *l* in the postoperative mGPS = 2 and mGPS < 2 groups, respectively. For multicategorical variables, the maximum absolute level-specific SMD was reported.

Pearson correlation coefficients were calculated as

$$r_{\;jk}=\frac{\sum_{i=1}^{n}(x_{ij}-{\bar{x}}_{j})(x_{ik}-{\bar{x}}_{k})}{\sqrt{\sum_{i=1}^{n}{\left(x_{ij}-{\bar{x}}_{j}\right)}^{2}}\sqrt{\sum_{i=1}^{n}{\left(x_{ik}-{\bar{x}}_{k}\right)}^{2}}},$$ (5)

where $x_{ij}$ and $x_{ik}$ denote the values of variables *j* and *k* for patient *i*.

For model evaluation, predicted probabilities were denoted as ${\hat{\;p}}_{i}$. The Youden index used to determine the optimal classification threshold was defined as

J=Sensitivity+Specificity−1. (6)

At the Youden-optimised threshold, the confusion matrix metrics were calculated as follows:

$$\mathrm{Sensitivity}=\frac{TP}{TP+FN},\;\mathrm{Specificity}=\frac{TN}{TN+FP},$$ (7)

$$\begin{array}{rl} \mathrm{Accuracy} & =\frac{TP+TN}{TP+TN+FP+FN},\mathrm{Precision}=\mathrm{PPV}\\ & =\frac{TP}{TP+FP}, \end{array}$$ (8)

$$\mathrm{NPV}=\frac{TN}{TN+FN},F_{1}=\frac{2\times \mathrm{Precision}\times \mathrm{Sensitivity}}{\mathrm{Precision}+\mathrm{Sensitivity}}.$$ (9)

Cohen's κ was calculated as.

$$\kappa =\frac{\;p_{o}-p_{e}}{1-p_{e}},$$ (10)

where $p_{o}$ is the observed agreement and $p_{e}$ is the expected agreement by chance.

The Brier score was calculated as the mean squared difference between predicted probabilities and observed outcomes:

$$\mathrm{Brier}=\frac{1}{n}\overset{n}{\underset{i=1}{\sum }}({\hat{\;p}}_{i}-Y_{i}{)}^{2}.$$ (11)

Decision curve analysis was used to estimate net benefit across threshold probabilities. For a given threshold probability $p_{t}$, net benefit was defined as

$$\mathrm{Net}\;\mathrm{Benefit}(\;p_{t})=\frac{TP}{n}-\frac{FP}{n}\times \frac{\;p_{t}}{1-p_{t}}.$$ (12)

For the SuperLearner framework, the final ensemble prediction was calculated as a weighted combination of base-learner predicted probabilities:

$${\hat{\;p}}_{i}^{SL}=\overset{M}{\underset{m=1}{\sum }}w_{m}{\hat{\;p}}_{im},\overset{M}{\underset{m=1}{\sum }}w_{m}=1,$$ (13)

where ${\hat{\;p}}_{im}$ is the predicted probability from base learner *m* for patient *i*, and $w_{m}$ denotes the corresponding model weight. For the NNLS-based SuperLearner-W, non-negative weights were imposed. For the Ridge-based SuperLearner-SR, weights were estimated under L2 regularisation.

For SHAP-based interpretation, the model output for an individual patient was decomposed as

$$f(x_{i})=E[\;f(X)]+\overset{p}{\underset{\;j=1}{\sum }}\phi_{ij},$$ (14)

where E[f(X)] denotes the SHAP reference output associated with the explainer background, $\phi_{ij}$ is the SHAP value of feature *j* for patient *i*, and *p* is the number of predictors. The quantities E[f(X)], $\phi_{ij}$ and $f(x_{i})$ are reported on the output scale used by the SHAP explainer. Accordingly, $f(x_{i})$ represents the explained model output rather than the calibrated predicted probability. Patient-level predicted probabilities were obtained separately from the TabICLv2 predict_proba output.

### 2.8 Development of the web-based prediction platform

To facilitate research-stage model demonstration and interpretation, the representative TabICLv2 model selected for downstream interpretation, together with the SHAP interpretability module, was incorporated into an interactive web-based application. The front-end user interface and back-end inference logic were developed using the open-source Streamlit framework in Python. The application was deployed on a cloud server to enable real-time risk probability estimation and individualised SHAP visualisation based on user-inputted preoperative baseline parameters.

For the web implementation, local SHAP values were recalculated on the positive-class probability scale using the mean feature profile of the training cohort as the explainer background, allowing the SHAP decomposition to reconstruct the displayed patient-level predicted probability directly.

To reduce unqualified extrapolation of population-level predictions to individual patients, an applicability-domain assessment was additionally incorporated into the web-based research prototype. The applicability-domain reference was derived exclusively from the final training cohort using the eight predictors retained for model development. For each predictor, the observed minimum and maximum values in the training cohort were recorded to characterise univariate data support. Multivariable similarity was additionally assessed using the Mahalanobis distance of the eight-dimensional feature profile from the training-cohort reference distribution. The covariance matrix was estimated from the training data, with its Moore–Penrose pseudoinverse used for numerical stability. The empirical 95th percentile of the training-cohort Mahalanobis-distance distribution was used as the predefined multivariable threshold. A patient was flagged as having limited training-data support when at least one predictor fell outside its observed training range or when the multivariable Mahalanobis distance exceeded this threshold. This applicability-domain assessment was intended to identify potentially under-represented feature profiles and should not be interpreted as a formal prediction interval or calibrated measure of predictive uncertainty.

## 3 Results

### 3.1 Patient baseline characteristics

The final analysis included 1,196 patients who underwent elective radical resection for colorectal cancer (CRC). Within this cohort, 507 patients (42.4%) developed postoperative mGPS = 2 during the acute postoperative phase, whereas the remaining 689 patients (57.6%) did not develop postoperative mGPS = 2 and formed the comparison group (mGPS < 2). Baseline characteristics between the two groups were generally balanced regarding sex distribution, body temperature and major comorbidities, including hypertension and diabetes mellitus, as reflected by small absolute standardised mean differences (SMDs).

However, inter-group differences were observed in demographic profiles and baseline laboratory indices. Clinically, patients in the mGPS = 2 group were significantly older and showed differences in primary tumour anatomical location. Regarding haematological and biochemical parameters, the mGPS = 2 group displayed lower preoperative hepatic synthetic and nutritional reserve, reflected by significantly lower levels of prealbumin (PA), cholinesterase (ChE), albumin (ALB), serum calcium (Ca) and haemoglobin (Hb). Baseline immune status, reflected by lymphocyte percentage (Lymph%) and absolute lymphocyte count, was also lower in these patients.

In addition to immune and nutritional impairment, patients who developed postoperative mGPS = 2 showed a more pronounced preoperative systemic inflammatory and hypercoagulable profile. This was characterised by elevated inflammatory and pro-thrombotic markers, including C-reactive protein (CRP), fibrinogen (Fbg) and D-dimer. Circulating tumour markers, including carcinoembryonic antigen (CEA) and carbohydrate antigen 125 (CA125), were also significantly higher in the mGPS = 2 group. Baseline platelet count (PLT) showed no significant difference between the two cohorts in univariate analysis and had a small absolute SMD, although it emerged as a nonlinear predictor in subsequent machine learning models.

Key baseline characteristics, including *P* values and absolute SMDs, are summarised in Table 1, and the complete baseline comparison is provided in Supplementary Table S1.

### 3.2 Feature dimensionality reduction and core predictor subset identification

To reduce the influence of multicollinearity within the high-dimensional feature space, Pearson correlation analysis incorporating Benjamini–Hochberg false discovery rate (FDR) correction was initially performed across all continuous baseline variables (Supplementary Figure S1). Applying a correlation coefficient threshold of |r|>0.70 and an FDR-adjusted P<0.05, highly collinear redundant feature pairs were selectively removed based on clinical relevance and data completeness, such as retaining neutrophil percentage over absolute neutrophil count and preserving pulse rate while removing redundant heart rate variables. This preliminary filtering phase yielded 60 independent candidate features.

Subsequently, recursive feature elimination (RFE) was used to further reduce feature dimensionality. As shown in Supplementary Figure S2, sequential incorporation of candidate features based on their relative contributions generated an AUC trajectory characterised by an initial increase followed by a plateau. The RFE algorithm identified eight optimal core predictors that contributed most to model performance: prealbumin (PA), age, fibrinogen (Fbg), albumin (ALB), cholinesterase (ChE), lymphocyte percentage (Lymph%), platelet count (PLT) and serum calcium (Ca).

After these eight features were integrated, adding further variables produced limited performance gains and increased the risk of model complexity and overfitting. This non-redundant eight-variable subset was consistent with the principle of model parsimony and reflected clinically relevant dimensions associated with postoperative mGPS = 2, including hypercoagulability, impaired hepatic synthetic function, nutritional depletion and systemic immunosuppression. This streamlined feature subset was therefore used as the standard input matrix for model development and evaluation, including the eight conventional machine learning algorithms, the TabICLv2 foundation model, the intermediate stacking models and the SuperLearner ensemble frameworks.

### 3.3 Performance comparison and representative model selection

Hyperparameter optimisation for the conventional machine learning algorithms was performed using the Optuna framework with five-fold cross-validation (Supplementary Table S2). In contrast, the TabICLv2 foundation model leveraged its pre-trained in-context learning (ICL) architecture to generate predictive representations without extensive dataset-specific tuning.

Comprehensive performance metrics are provided in Supplementary Table S3, and receiver operating characteristic (ROC) curves are presented in Figure 2.

**Figure 2:**
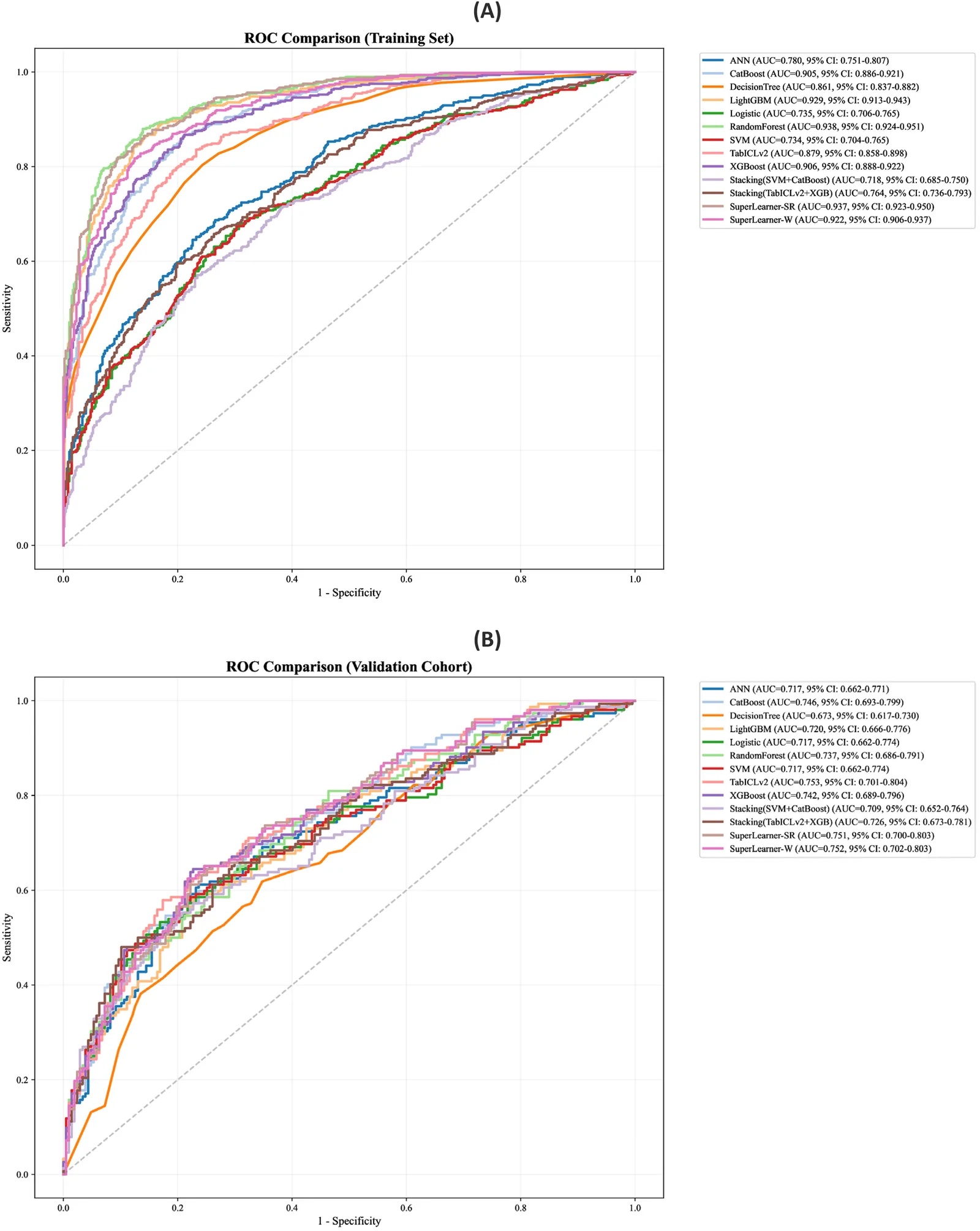
Receiver operating characteristic curves of the predictive models. **(A)** ROC curves in the training cohort. **(B)** ROC curves in the internal validation cohort. ROC, receiver operating characteristic; AUC, area under the curve; CI, confidence interval.

In the training cohort, tree-based ensemble models showed high apparent discrimination, including Random Forest (AUC = 0.938), LightGBM (AUC = 0.929) and CatBoost (AUC = 0.905). Their discrimination was lower in the independent internal validation cohort, with validation AUCs of 0.737 for Random Forest, 0.720 for LightGBM and 0.746 for CatBoost. Because a single training–validation performance difference does not by itself establish overfitting or generalisability, these differences were interpreted descriptively and were further examined using learning-curve analysis.

Learning-curve analysis revealed suboptimal convergence characteristics across the evaluated models (Supplementary Figure S8). Cross-validation performance showed only modest changes as the effective training-set size increased, and the cross-validation trajectories remained relatively flat overall. At the largest training fraction, the mean cross-validation AUC was 0.756 for TabICLv2, 0.739 for CatBoost, 0.737 for Random Forest, 0.735 for XGBoost and 0.726 for LightGBM. Although the separation between training and cross-validation AUC narrowed to varying degrees for TabICLv2, CatBoost and XGBoost, convergence was not achieved within the available training sample, and a more persistent separation remained for Random Forest and LightGBM. Thus, persistent training–cross-validation gaps and the limited incremental improvement in cross-validation performance indicate that stable and generalisable learning dynamics were not demonstrated within the available sample size. These findings represent an important limitation for clinical deployment, and the current models should be regarded as research-stage predictive tools rather than clinically deployable systems.

In the internal validation cohort, the standalone TabICLv2 model achieved the highest discrimination among the single models, with an AUC of 0.753 (95% CI, 0.701–0.804), followed by XGBoost (AUC = 0.742), Random Forest (AUC = 0.737) and Logistic Regression (AUC = 0.717). The SuperLearner frameworks showed comparable validation discrimination, with validation AUCs of 0.752 for SuperLearner-W and 0.751 for SuperLearner-SR. Given the small absolute differences in validation AUC among the top-performing models, these comparisons should be interpreted descriptively rather than as evidence of statistical superiority.

Precision–recall analysis provided complementary assessment of model discrimination in the internal validation cohort (Supplementary Figure S4). XGBoost achieved the highest average precision (AP = 0.697), followed by Random Forest (AP = 0.693), CatBoost (AP = 0.692), TabICLv2 (AP = 0.690) and SuperLearner-W (AP = 0.690); SuperLearner-SR achieved an AP of 0.688. These values were all above the no-skill reference defined by the validation-cohort event prevalence (0.423). The relatively small differences among the leading models further indicate that no single model consistently dominated across all discrimination metrics.

For downstream interpretation and web-based implementation, TabICLv2 was selected as the representative model using the multidimensional considerations described above. It achieved the numerically highest validation AUC (0.753), shared the lowest Brier score with SuperLearner-W (0.197), and showed favourable net benefit on decision curve analysis. In addition, the SuperLearner weighting patterns indicated that TabICLv2 provided the dominant contribution to both ensembles, accounting for 77.9% of the weight in SuperLearner-W and 77.7% in SuperLearner-SR. Given that TabICLv2 could also be deployed directly as a standalone model without an additional meta-learning layer, it was considered the most practical representative model for SHAP interpretation and prototype deployment. This selection should not be interpreted as evidence of statistically superior predictive performance relative to the other leading models.

After determining the optimal classification probability threshold by maximising the Youden index, the TabICLv2 model achieved a specificity of 0.826 and an accuracy of 0.721 for predicting postoperative mGPS = 2 in the internal validation cohort. The positive predictive value (PPV) and negative predictive value (NPV) were 0.710 and 0.728, respectively, with an F1 score of 0.638 and Cohen's κ of 0.415. These threshold-dependent metrics characterise the internal validation performance of the model and should not be interpreted as evidence of readiness for clinical deployment.

The threshold-dependent sensitivity of several models was moderate rather than high. This should be interpreted in the context of the operating-threshold selection procedure: thresholds were selected from out-of-fold predictions in the training cohort by maximising the Youden index, which jointly balances sensitivity and specificity rather than explicitly prioritising sensitivity. For example, TabICLv2 used an operating threshold of 0.525 and achieved a sensitivity of 0.579 together with a specificity of 0.826 in the internal validation cohort. Random Forest showed the highest sensitivity (0.809), but this was obtained at a substantially lower operating threshold of 0.361 and was accompanied by a markedly lower specificity of 0.536 and positive predictive value of 0.562. Thus, the higher sensitivity of Random Forest reflects a different threshold-specific sensitivity–specificity trade-off rather than superior overall discrimination or clinical performance.

### 3.4 Model calibration and decision-curve analysis

Calibration plots (Supplementary Figure S3) illustrated the agreement between predicted and observed probabilities for each model in the internal validation cohort. Overall, the better-performing models showed calibration curves that approximated the ideal diagonal reference line, suggesting acceptable probability calibration.

The TabICLv2 foundation model and SuperLearner-W showed the lowest Brier score (0.197), followed by SuperLearner-SR (Brie*r* = 0.198), CatBoost (Brie*r* = 0.199) and XGBoost (Brie*r* = 0.200). These results indicate that the predicted probabilities generated by TabICLv2 and the SuperLearner models were generally consistent with the observed frequency of postoperative mGPS = 2. In contrast, the Decision Tree model showed poorer calibration performance (Brie*r* = 0.259).

Decision curve analysis (DCA) is presented in Figure 3. Across a range of clinically relevant threshold probabilities, TabICLv2 and the SuperLearner ensembles provided greater net benefit than several conventional standalone machine learning algorithms. These findings indicate favourable decision-analytic net benefit relative to several comparator models within the internal validation setting; however, they do not establish clinical utility or readiness for clinical deployment. Threshold-specific confusion matrices for the five leading models are presented in Figure 4. At the training-derived Youden thresholds, TabICLv2 yielded 88 true-positive (TP), 171 true-negative (TN), 36 false-positive (FP) and 64 false-negative (FN) classifications. SuperLearner-W yielded TP = 95, TN = 162, FP = 45 and FN = 57, whereas SuperLearner-SR yielded TP = 98, TN = 155, FP = 52 and FN = 54. CatBoost yielded TP = 80, TN = 175, FP = 32 and FN = 72, while XGBoost yielded TP = 98, TN = 160, FP = 47 and FN = 54. These results demonstrate that models with similar overall discrimination may nevertheless exhibit different sensitivity–specificity trade-offs at their selected operating thresholds.

**Figure 3:**
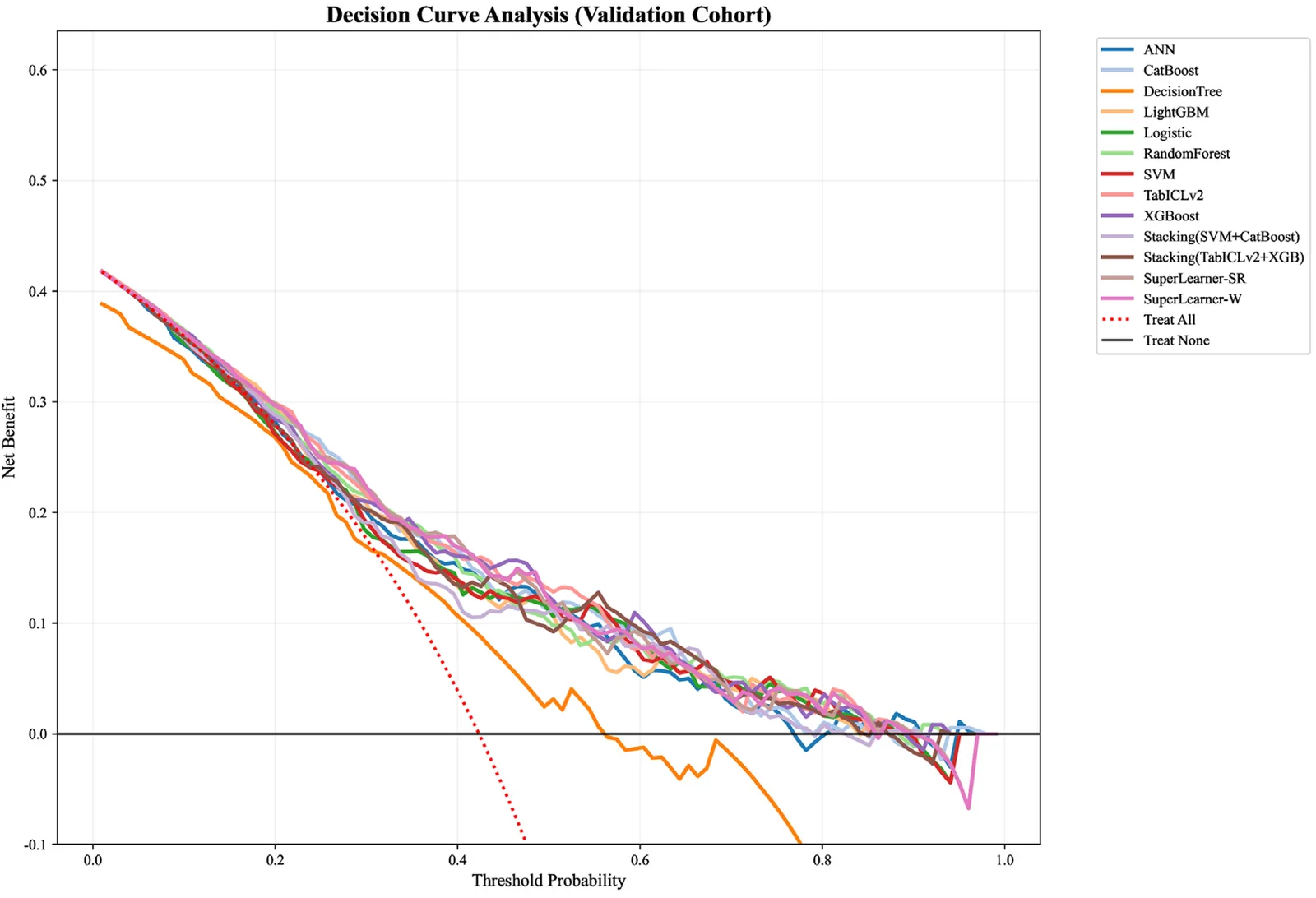
Decision curve analysis of the predictive models in the internal validation cohort. DCA, decision curve analysis; mGPS, modified Glasgow Prognostic Score.

**Figure 4:**
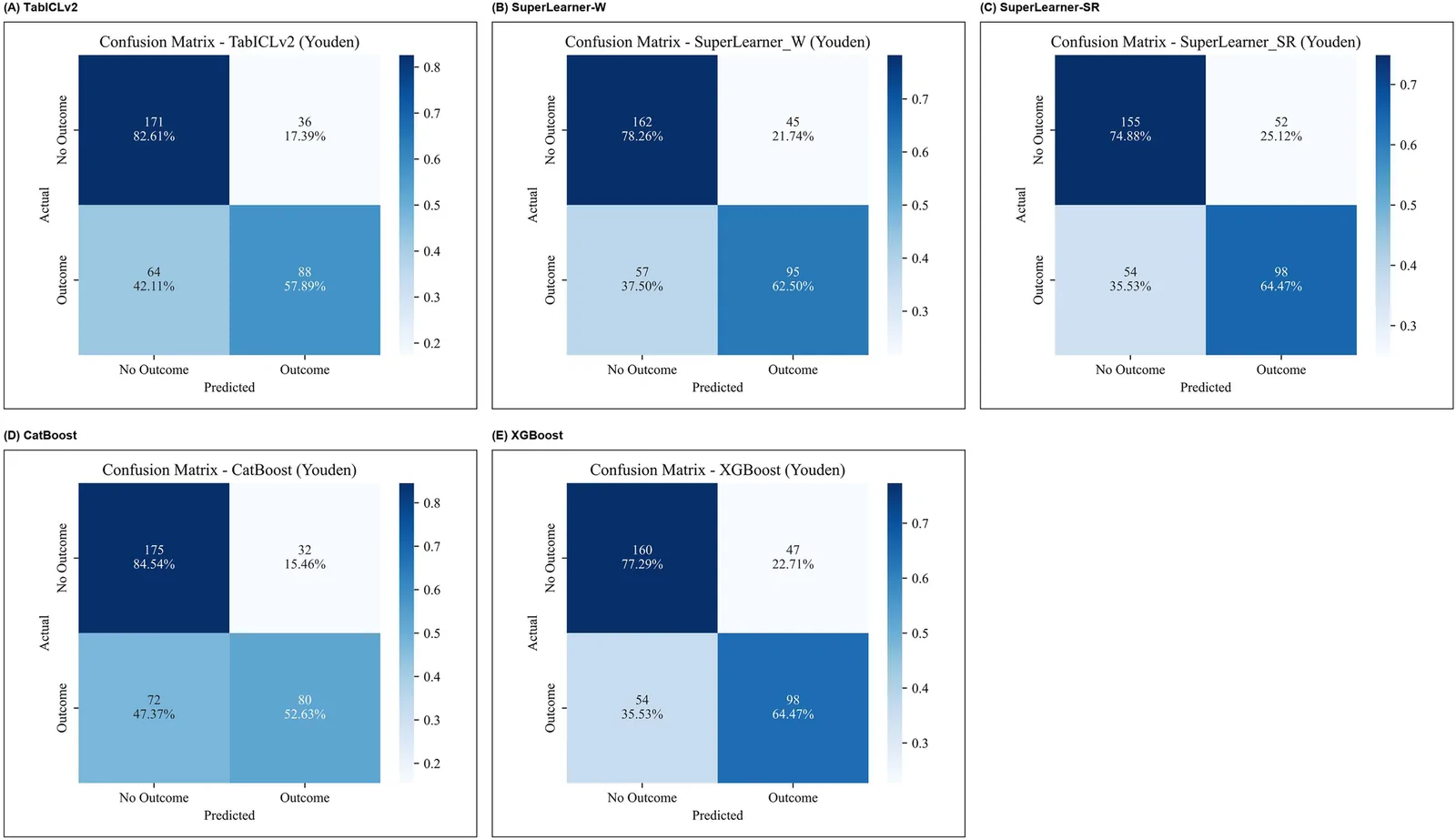
Confusion matrices of the five leading predictive models in the internal validation cohort using model-specific operating thresholds derived from out-of-fold predictions in the training cohort by maximising the Youden index. **(A)** TabICLv2; **(B)** SuperLearner-W; **(C)** SuperLearner-SR; **(D)** CatBoost; and **(E)** XGBoost. Rows represent the actual outcome category and columns represent the predicted category. Values in each cell indicate the number of patients and the corresponding percentage within the actual outcome category. “Outcome” indicates postoperative mGPS = 2, whereas “No Outcome” indicates postoperative mGPS < 2. mGPS, modified Glasgow Prognostic Score.

### 3.5 Model interpretability and clinical feature attribution

Following its selection as the representative model for downstream interpretation, SHAP (SHapley Additive exPlanations) analysis was performed to improve interpretability and clarify the decision-making rationale of the TabICLv2 model.

The global SHAP beeswarm plot (Figure 5) identified lymphocyte percentage (Lymph%), platelet count (PLT) and fibrinogen (Fbg) as the three leading contributors to the prediction of postoperative mGPS = 2. Lower values of Lymph%, albumin (ALB), prealbumin (PA), cholinesterase (ChE) and serum calcium (Ca) were generally associated with higher SHAP values, indicating an increased model output towards postoperative mGPS = 2. Conversely, higher values of Fbg, PLT and age also contributed positively to the model output. These findings suggest that immune suppression, hypercoagulable inflammation, impaired hepatic synthetic function and nutritional depletion may jointly contribute to the development of postoperative mGPS = 2.

**Figure 5:**
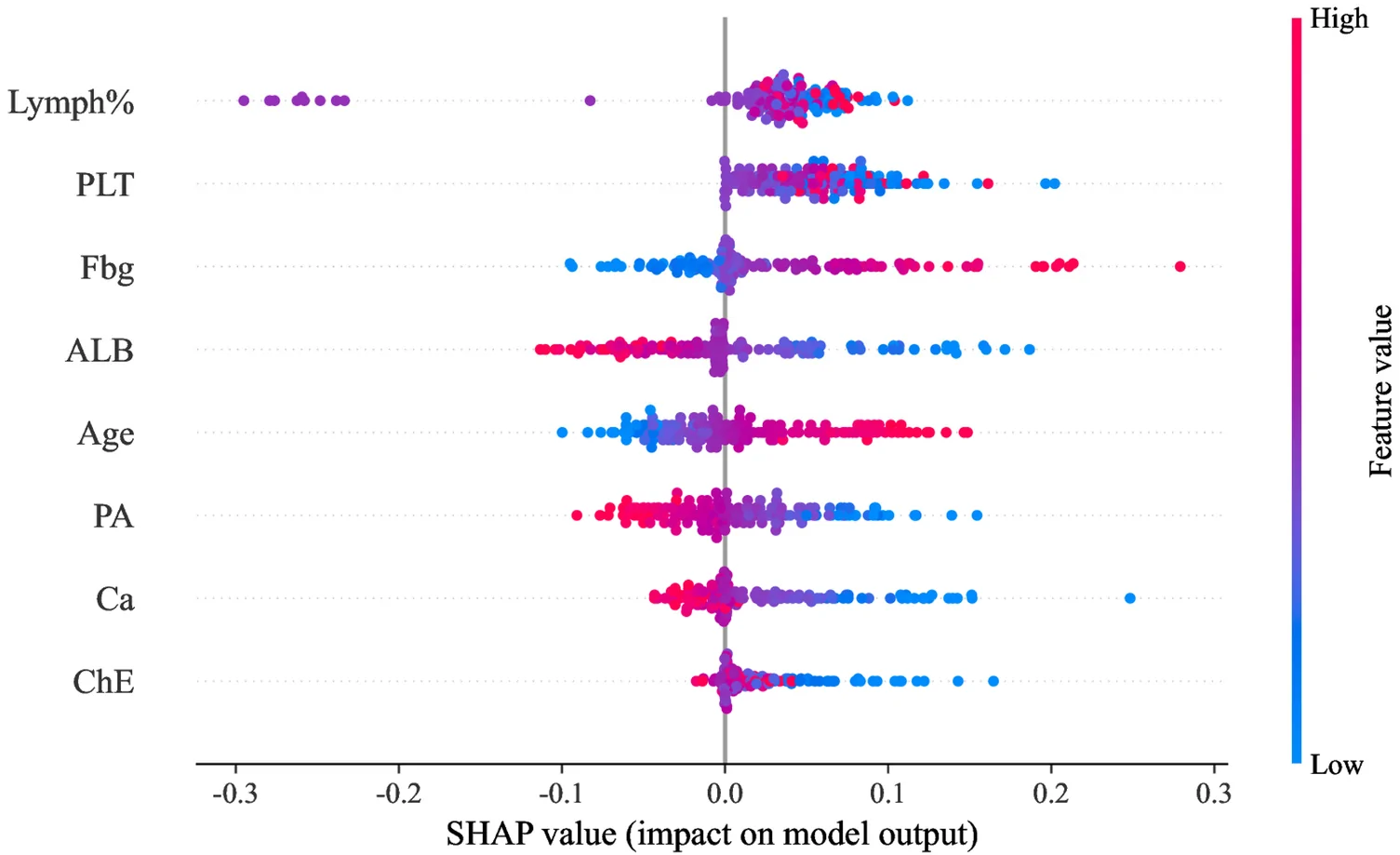
Global SHAP beeswarm plot of the TabICLv2 model showing feature importance and directional effects on the prediction of postoperative mGPS = 2. SHAP, SHapley Additive exPlanations; mGPS, modified Glasgow Prognostic Score; Lymph%, lymphocyte percentage; PLT, platelet count; Fbg, fibrinogen; ALB, albumin; PA, prealbumin; Ca, serum calcium; ChE, cholinesterase.

SHAP dependence plots with LOWESS smoothing curves (Supplementary Figure S5) further illustrated nonlinear patterns for the core predictors. Increased SHAP contributions were observed when preoperative ALB was below approximately 38 g/L, PA below 200 mg/L or ChE below 6,000 U/L. Similarly, increased model output towards postoperative mGPS = 2 was observed when Fbg exceeded approximately 3.0 g/L or when age was above approximately 65 years. A lymphocyte percentage below approximately 20% was also associated with an increased SHAP contribution towards postoperative mGPS = 2 prediction.

To translate population-level patterns into individual-level interpretation, local SHAP waterfall plots were generated for illustrative high- and low-risk patients (Supplementary Figures S6A and S6B). For the high-risk patient, positive feature contributions shifted the explained model output towards postoperative mGPS = 2, whereas for the low-risk patient, negative feature contributions shifted the output away from postoperative mGPS = 2. The displayed E[f(X)] and f(x) values are on the SHAP explainer's output scale and were not interpreted as calibrated probabilities. The probability-based selection of the illustrative patients was performed separately using predicted probabilities from TabICLv2.

### 3.6 Development of a web-based prediction platform and research-stage implementation

To support research-stage model demonstration and transparent interpretation, a dynamic web-based research prototype driven by the representative TabICLv2 model selected for downstream interpretation was developed and deployed at https://mgps-tabicl-model.streamlit.app. The platform is intended exclusively for research and demonstration purposes and should not be interpreted as a clinically validated decision-support system. To improve accessibility and reproducibility, the source code and deployment instructions for the web-based research prototype have additionally been made publicly available through GitHub at https://github.com/cxk565/postoperative-mgps-tabicl-calculator.

The platform integrates the eight RFE-selected predictors: prealbumin (PA), age, fibrinogen (Fbg), albumin (ALB), cholinesterase (ChE), lymphocyte percentage (Lymph%), platelet count (PLT) and serum calcium (Ca). After entering a patient's routine preoperative laboratory parameters into the interactive interface, users can obtain an estimated probability of postoperative mGPS = 2.

The calculator also incorporates a local interpretability module. The platform generates individualised SHAP-based attribution graphics to help users understand which variables, such as hypercoagulability, immunosuppression or impaired nutritional reserve, contribute to a specific patient's predicted risk and in which direction. This design may help reduce concerns regarding the black-box nature of artificial intelligence (AI) models by providing transparent visualisation of patient-level model attribution within the research prototype.

The platform further incorporated an applicability-domain assessment to indicate how well an individual patient's feature profile was represented by the model-development data. The reference distribution was derived exclusively from the final training cohort. The module identifies individual predictor values falling outside the observed training ranges and evaluates the joint eight-variable profile using its Mahalanobis distance relative to the training-cohort distribution. Profiles exceeding the empirical 95th-percentile Mahalanobis-distance threshold, or containing at least one predictor value outside its observed training range, are flagged as having limited training-data support, with a warning that the resulting prediction should be interpreted with additional caution. This applicability-domain assessment reflects distributional support from the model-development population and should not be interpreted as a formal predictive uncertainty interval or as a guarantee of prediction accuracy.

The web-based calculator was developed strictly as a research prototype for model demonstration and interpretation. No patient-identifiable information should be entered into the platform. The calculator must not be used for clinical decision-making, patient care, treatment selection or other real-world clinical purposes in its current form.

#### 3.6.1 Illustrative application example

A representative preoperative scenario is shown in Supplementary Figure S7 to illustrate the output of the web-based calculator. The input parameters for this example were as follows: age = 76.5 years, prealbumin (PA) = 55 mg/L, albumin (ALB) = 26.3 g/L, cholinesterase (ChE) = 1,218 U/L, lymphocyte percentage (Lymph%) = 17.5%, platelet count (PLT) =  $253\times 10^{9}$/L, fibrinogen (Fbg) = 3.37 g/L and serum calcium (Ca) = 2.02 mmol/L.

Based on these inputs, the model estimated a predicted probability of 96.16% for postoperative mGPS = 2. The corresponding real-time SHAP waterfall plot, calculated on the positive-class probability scale using the mean feature profile of the model-development cohort as the reference, identified low ALB, markedly reduced ChE, decreased Ca and low PA as the principal positive contributors to the elevated predicted risk. This example illustrates how the calculator provides numerical risk estimation and patient-level feature attribution within the research prototype; however, the output must not be used for clinical decision-making, patient care or other real-world clinical purposes.

## 4 Discussion

This study developed and internally validated predictive models for postoperative mGPS = 2 in patients with colorectal cancer (CRC). Following dimensionality reduction via recursive feature elimination (RFE), eight core preoperative predictors were identified: prealbumin (PA), age, fibrinogen (Fbg), albumin (ALB), cholinesterase (ChE), lymphocyte percentage (Lymph%), platelet count (PLT) and serum calcium (Ca). Several modelling approaches achieved closely comparable discrimination in the internal validation cohort. Importantly, learning-curve analysis revealed suboptimal convergence behaviour across the evaluated models. Cross-validation performance remained relatively flat across increasing training fractions, while persistent separation from training performance remained even at the largest training fraction. Although the magnitude of this separation decreased for some models, satisfactory convergence was not demonstrated within the available training sample. These characteristics do not demonstrate the stable learning dynamics expected for a model intended for clinical deployment and raise concerns regarding model generalisability and reliability. Therefore, despite the moderate discrimination observed in internal validation, the current models are not ready for clinical deployment. TabICLv2 was retained as the representative model for downstream interpretation and research-prototype implementation on the basis of its discrimination, calibration, decision-curve performance and simpler standalone deployment, rather than as a statistically superior or clinically validated model.

The tissue trauma induced by CRC surgery and the subsequent systemic inflammatory response (SIR) are critical to the postoperative recovery trajectory. Previous studies have indicated that the surgical approach, such as open, laparoscopic or robot-assisted surgery, and anaesthetic management strategies may significantly alter the magnitude of the postoperative SIR, thereby influencing short- and long-term outcomes (25–27). Recent machine-learning studies provide additional evidence that procedural characteristics are important components of postoperative risk. Anania et al. identified intraoperative bleeding, transfusion status, anastomotic technique and fast-track adherence among relevant predictors of complications following laparoscopic right hemicolectomy for colon cancer (19). Elengickal et al. further demonstrated that operative duration should not necessarily be considered independently of surgical approach; their combined procedural phenotype analysis identified prolonged open surgery as the most influential predictor of postoperative complications (21). Similarly, Du et al. found that surgical approach, intraoperative hypothermia, acidosis and hypoxaemia, together with inflammatory markers, contributed to postoperative sepsis prediction after emergency colon cancer surgery (22). These findings emphasise that perioperative outcomes arise from an interaction between baseline biological vulnerability and the subsequent procedural insult. Accordingly, because the present model deliberately uses preoperative variables only, it should be interpreted primarily as an estimate of preoperative susceptibility to postoperative inflammatory–nutritional deterioration rather than as a comprehensive model of total perioperative risk. Through SHAP attribution analysis, this study identified Lymph%, PLT and Fbg as important predictive indicators associated with postoperative mGPS = 2 in patients with CRC. The imbalance between platelet count and lymphocyte status may reflect an immunosuppressive and pro-inflammatory state, consistent with the systemic immune-inflammatory burden reported in studies using indices such as SII and NLR in CRC (28–30). Concurrently, elevated fibrinogen indicates a preoperative hypercoagulable and hyperinflammatory state; when exposed to surgical stress, such patients may be more susceptible to an excessive inflammatory response.

Hepatic synthetic function and nutritional indicators, represented by ALB, PA and ChE, also contributed substantially to the model. Preoperative nutritional and metabolic deterioration is not only a direct manifestation of chronic CRC-related catabolic consumption but may also reduce stress tolerance and increase vulnerability to postoperative complications. This finding is consistent with previous studies showing that prognostic models incorporating nutritional metrics can predict CRC outcomes (31, 32). Meanwhile, advanced age represents a non-modifiable risk factor, and its accompanying physiological frailty may increase the risk of severe postoperative complications and organ decompensation (33, 34). The local SHAP waterfall plots in this study further illustrated that advanced age combined with depleted immune and nutritional reserves may act as an important risk-amplifying pattern for postoperative inflammatory–nutritional deterioration in CRC.

Compared with previous predictive systems relying heavily on single biomarkers or conventional linear logistic regression, the TabICLv2 model and SuperLearner ensemble architecture used in this study may offer methodological advantages. Recently, ensemble machine learning models combined with SHAP-based explanatory frameworks have been applied to the assessment of CRC recurrence, postoperative heart failure, lymph node metastasis and other acute clinical conditions (35–41). Similarly, the introduction of SHAP in this study helped reduce the black-box barrier of artificial intelligence by providing transparent, patient-level individualised feature attribution, thereby improving the transparency and interpretability of the model outputs.

In terms of research-stage translation, the web-based application developed in this study should be regarded strictly as a research prototype. Although the platform demonstrates how individualised probability estimates, SHAP-based feature attribution and applicability-domain information can be presented in an accessible format, the learning-curve findings indicate that stable and generalisable learning dynamics have not yet been established. Accordingly, the calculator must not be used for clinical decision-making, patient care, treatment selection or other real-world clinical purposes at the current stage. Further model development, independent external validation and prospective evaluation are required before any clinical implementation can be considered.

This study has several limitations. First, as a single-centre retrospective cohort study, potential selection bias and residual confounding could not be entirely eliminated. Second, although internal validation was performed using a stratified training-validation split and all modelling procedures were derived from the training cohort, external validation across multicentre CRC cohorts from diverse geographical regions is required to confirm generalisability (42). Third, the outcome was defined using routinely available postoperative CRP and albumin measurements during the first postoperative week; therefore, variations in postoperative testing frequency, perioperative care pathways and nutritional or anti-inflammatory interventions may have influenced endpoint ascertainment. Fourth, the model was based only on preoperative variables. Intraoperative and early postoperative factors, such as operative time, blood loss, surgical approach and postoperative complications, were not incorporated in the current model.

Fifth, learning-curve analysis revealed suboptimal convergence characteristics. Cross-validation performance remained relatively flat across increasing training fractions, and persistent separation between training and cross-validation performance remained even at the largest training fraction. Although this gap narrowed to varying degrees for some models, satisfactory convergence was not demonstrated within the available sample size. These findings suggest that stable and generalisable learning dynamics have not yet been established and fundamentally limit the readiness of the models for clinical deployment. Accordingly, the models and the associated web-based tool cannot currently be recommended for real-world clinical use.

Sixth, population-level predictive performance does not ensure equally reliable predictions for every individual patient. Prediction reliability may decrease when a patient's feature profile is sparsely represented in the model-development data. To provide a pragmatic safeguard against obvious extrapolation, the web-based prototype incorporates an applicability-domain assessment derived exclusively from the training cohort, combining observed predictor ranges with multivariable Mahalanobis distance. However, this procedure indicates only the degree of distributional support and does not constitute formal uncertainty quantification or guarantee the accuracy of an individual prediction. More rigorous uncertainty-quantification approaches, together with independent external and prospective validation, should therefore be evaluated in future studies.

Finally, the web-based calculator should be regarded strictly as a research prototype rather than a validated clinical decision-support system. In addition to the suboptimal learning-curve convergence described above, the present study did not include a formal clinician-centred evaluation of the platform's usability, interpretability, acceptability or potential influence on clinical decision-making. The calculator must therefore not be used for clinical decision-making or patient care in its current form. Further model development should aim to demonstrate more stable and generalisable learning behaviour, followed by independent external and prospective validation and structured clinician-centred evaluation, before implementation in routine clinical practice can be considered.

In conclusion, this study developed and internally validated an interpretable model to support preoperative risk stratification for postoperative inflammatory–nutritional deterioration after colorectal cancer surgery. Using eight routinely available preoperative variables, the model achieved moderate discrimination and captured a high-risk profile characterised by impaired immune, coagulation and nutritional reserve. SHAP analysis provided global and patient-level feature attribution, while the web-based calculator demonstrated the potential application of the model as a research-stage risk stratification tool. However, the suboptimal convergence observed in learning-curve analysis indicates that stable and generalisable learning dynamics have not yet been established. The model and web-based calculator should therefore remain strictly research tools and should not be used for clinical decision-making or patient care without further model development, independent external validation and prospective assessment.

## Data Availability

The datasets generated and analysed during the current study are not publicly available due to institutional data protection regulations and patient privacy restrictions but may be available from the corresponding author upon reasonable request and with permission from Wuxi Medical Center, Nanjing Medical University. The source code and deployment instructions for the webbased research prototype are publicly available at https://github.com/cxk565/postoperative-mgps-tabicl-calculator. Patient-level clinical data and serialised model objects are not included in the public repository because of institutional data-protection and patient- privacy requirements. Additional code related to model development and statistical analysis may be available from the corresponding author upon reasonable request, subject to institutional approval.
