# Supplementary material for: Preoperative risk stratification for postoperative inflammatory–nutritional deterioration after colorectal cancer surgery: an interpretable machine learning study
Source: Front Med (Lausanne). 2026 Sep 29;13:1938414. doi: 10.3389/fmed.2026.1938414 (PMC13658502; doi:10.3389/fmed.2026.1938414)
Supplement: Supplementary file 1 [file Supplementaryfile1.pdf]

Supplementary Material

SUPPLEMENTARY TABLES

Table S1: Complete baseline clinical and laboratory characteristics of the study population according to postoperative mGPS status.

Characteristics	mGPS = 2 ($n = 507$)	mGPS < 2 ($n = 689$)	P value	SMD
Sex, n (%)			0.885	0.008
Female	201 (39.6%)	276 (40.1%)		
Male	306 (60.4%)	413 (59.9%)		
Diagnosis, n (%)			0.004	0.169
Colon cancer	305 (60.2%)	357 (51.8%)		
Rectal cancer	202 (39.8%)	332 (48.2%)		
Hypertension, n (%)			0.221	0.072
No	289 (57.0%)	417 (60.5%)		
Yes	218 (43.0%)	272 (39.5%)		
Diabetes, n (%)			0.362	0.053
No	433 (85.4%)	601 (87.2%)		
Yes	74 (14.6%)	88 (12.8%)		
Age, median (IQR)	70.83 (62.25, 77.41)	64.58 (57.25, 71.33)	< 0.001	0.500
Temperature, median (IQR)	36.6 (36.5, 36.8)	36.6 (36.5, 36.8)	0.405	0.053
Pulse, median (IQR)	78 (74.5, 86)	78 (74, 83)	0.024	0.158
Diastolic pressure, median (IQR)	78 (70, 81)	80 (73, 83)	0.028	0.126
Systolic pressure, median (IQR)	130 (120, 140)	130 (120, 140)	0.628	0.005
Mono%, median (IQR)	7.6 (6.2, 9)	7.5 (6.2, 8.2)	0.003	0.205
WBC, median (IQR)	6.54 (5.21, 8.13)	6.81 (5.59, 7.58)	0.779	0.138
RBC, median (IQR)	4.24 (3.87, 4.655)	4.37 (4.16, 4.71)	< 0.001	0.321
MCV, median (IQR)	89.8 (84.35, 93.45)	89.2 (87, 92.7)	0.837	0.091
MCHC, median (IQR)	325 (314, 335)	327 (321, 336)	0.001	0.211
MCH, median (IQR)	29.5 (27.2, 30.9)	29.5 (28.5, 30.9)	0.156	0.149
RDW-CV, median (IQR)	13.2 (12.5, 14.45)	13 (12.4, 13.7)	< 0.001	0.236

Continued on next page

Characteristics	mGPS = 2 ($n = 507$)	mGPS $<$ 2 ($n = 689$)	P value	SMD
Lymph%, median (IQR)	23.7 (17.85, 31.05)	25.7 (21.9, 31.4)	$<$ 0.001	0.249
Lymph, median (IQR)	1.55 (1.19, 1.925)	1.67 (1.37, 2)	$<$ 0.001	0.211
Mono, median (IQR)	0.5 (0.38, 0.63)	0.48 (0.38, 0.55)	0.002	0.263
Neut%, median (IQR)	65.3 (57.45, 72.4)	64.3 (57.9, 68.9)	0.004	0.177
Neut, median (IQR)	4.23 (3.125, 5.59)	4.25 (3.25, 4.96)	0.329	0.180
Hct, median (IQR)	38.3 (33.4, 42.1)	39.1 (37, 42.9)	$<$ 0.001	0.316
Eos%, median (IQR)	1.6 (0.7, 2.8)	1.8 (0.9, 2.6)	0.148	0.018
Baso%, median (IQR)	0.4 (0.2, 0.6)	0.4 (0.3, 0.6)	0.012	0.111
Eos, median (IQR)	0.1 (0.04, 0.18)	0.11 (0.06, 0.16)	0.245	0.033
Baso, median (IQR)	0.03 (0.01, 0.04)	0.03 (0.02, 0.04)	0.017	0.060
Hb, median (IQR)	125 (107, 140)	129 (120, 143)	$<$ 0.001	0.316
PLT, median (IQR)	237 (190, 301)	247 (204, 281)	0.959	0.072
MPV, median (IQR)	10.3 (9.7, 11.1)	10.5 (9.9, 11.1)	0.057	0.090
PDW, median (IQR)	11.8 (10.45, 13.3)	12.2 (10.8, 13.5)	0.017	0.086
Platelet volume, median (IQR)	0.25 (0.2, 0.3)	0.26 (0.22, 0.29)	0.552	0.036
ALT, median (IQR)	13.6 (10, 19)	16.2 (11.9, 21.5)	$<$ 0.001	0.135
AST, median (IQR)	14 (7, 20)	14 (6, 19)	0.480	0.073
GGT, median (IQR)	22 (15, 37)	23 (16, 34)	0.541	0.110
AST/ALT, median (IQR)	1.48 (1.21, 1.78)	1.34 (1.05, 1.55)	$<$ 0.001	0.429
TBIL, median (IQR)	12.5 (9.3, 16)	12.9 (10.2, 15.5)	0.216	0.069
DBIL, median (IQR)	3.7 (2.8, 5.2)	3.8 (2.9, 4.8)	0.853	0.120
ALB, median (IQR)	38.5 (35.5, 40.6)	39.8 (38.8, 43.1)	$<$ 0.001	0.634
GLO, median (IQR)	28.5 (25.65, 31.5)	28.4 (25.5, 29.8)	0.008	0.177
TP, median (IQR)	67.5 (61.5, 71.7)	68 (64.9, 72.7)	$<$ 0.001	0.260
ALB/GLO, median (IQR)	1.38 (1.21, 1.49)	1.43 (1.35, 1.6)	$<$ 0.001	0.574
PA, median (IQR)	196.8 (150.4, 234.6)	223.7 (199, 261.1)	$<$ 0.001	0.629
TBA, median (IQR)	3.4 (1.8, 5.6)	3.1 (1.7, 4.6)	0.011	0.177
Crea, median (IQR)	70.1 (60.1, 84.85)	70.4 (59.1, 82.9)	0.479	0.110
Urea, median (IQR)	5.3 (4.3, 6.4)	5.3 (4.5, 6)	0.957	0.093
UA, median (IQR)	298.9 (249.9, 351.7)	315.6 (276.2, 358.9)	$<$ 0.001	0.250

Continued on next page

Characteristics	mGPS = 2 ($n = 507$)	mGPS < 2 ($n = 689$)	P value	SMD
Urea/Crea, median (IQR)	0.073 (0.059, 0.0905)	0.074 (0.063, 0.09)	0.418	0.015
TC, median (IQR)	4.54 (4.01, 5.075)	4.67 (4.25, 5.14)	0.003	0.157
TG, median (IQR)	1.21 (0.92, 1.56)	1.31 (1.02, 1.63)	0.002	0.164
HDL-C, median (IQR)	1.08 (0.935, 1.245)	1.09 (0.97, 1.24)	0.064	0.127
LDL-C, median (IQR)	2.73 (2.395, 3.06)	2.79 (2.52, 3.06)	0.082	0.034
Lpa, median (IQR)	301 (193, 403.5)	290 (159, 348)	0.009	0.198
apoAI, median (IQR)	1.2 (1.05, 1.345)	1.25 (1.13, 1.38)	< 0.001	0.276
apoB, median (IQR)	0.87 (0.77, 0.98)	0.88 (0.78, 0.96)	0.690	0.080
K^+, median (IQR)	3.93 (3.64, 4.18)	3.92 (3.71, 4.1)	0.937	0.054
Na^+, median (IQR)	140.6 (138.9, 141.8)	140.6 (140, 141.9)	< 0.001	0.294
Cl^-, median (IQR)	105 (103, 106.9)	105 (104.1, 106.7)	0.035	0.201
Ca, median (IQR)	2.23 (2.145, 2.3)	2.27 (2.22, 2.36)	< 0.001	0.528
P, median (IQR)	1.14 (1.03, 1.235)	1.16 (1.06, 1.25)	0.025	0.092
Mg^{2+}, median (IQR)	0.87 (0.84, 0.9)	0.87 (0.84, 0.9)	0.267	0.091
Glu, median (IQR)	5.46 (4.79, 6.415)	5.37 (4.78, 6.05)	0.098	0.194
LDH, median (IQR)	182 (158, 210)	181 (156, 203)	0.250	0.131
ALP, median (IQR)	83 (70.5, 98)	82 (70, 92)	0.091	0.180
ADA, median (IQR)	9.2 (7.4, 11.2)	8.8 (7.1, 10.2)	< 0.001	0.239
ChE, median (IQR)	6405 (5264, 7358)	7048 (6280, 8059)	< 0.001	0.542
Cys-C, median (IQR)	0.88 (0.75, 1.03)	0.83 (0.73, 0.95)	< 0.001	0.254
FFA, median (IQR)	0.44 (0.32, 0.575)	0.45 (0.35, 0.56)	0.361	0.008
RBP, median (IQR)	33.7 (26.6, 42.05)	38.3 (32.7, 45.2)	< 0.001	0.435
CO_2CP, median (IQR)	27 (25.3, 28.6)	27 (25.9, 28.2)	0.740	0.035
eGFR, median (IQR)	86.9 (77.25, 95.25)	91.2 (82.8, 98.2)	< 0.001	0.332
Osm, median (IQR)	282 (279, 284)	282 (280, 284)	0.008	0.171
AFU, median (IQR)	14 (12, 16)	14 (12, 16)	0.256	0.055
AG, median (IQR)	8.4 (7.1, 9.5)	8.5 (7.5, 9.6)	0.105	0.008
PT-INR, median (IQR)	1.06 (1.01, 1.12)	1.05 (1, 1.09)	0.015	0.155
PT, median (IQR)	12 (11.4, 12.6)	11.8 (11.3, 12.3)	< 0.001	0.205
TT, median (IQR)	20.5 (19.2, 21.75)	20.6 (19.4, 21.6)	0.978	0.054

Continued on next page

Characteristics	mGPS = 2 ($n = 507$)	mGPS $<$ 2 ($n = 689$)	P value	SMD
ATIII:A, median (IQR)	90 (80, 101)	96 (87, 104)	$<$ 0.001	0.352
APTT, median (IQR)	29.9 (28, 32)	30.1 (28.4, 31.8)	0.254	0.060
Fbg, median (IQR)	3.21 (2.78, 3.75)	3.08 (2.71, 3.34)	$<$ 0.001	0.365
FDP, median (IQR)	1.52 (0.835, 3.685)	1.14 (0.66, 2.15)	$<$ 0.001	0.357
D-dimer, median (IQR)	192 (110.5, 429.5)	136 (83, 257)	$<$ 0.001	0.368
CRP, median (IQR)	3.1 (1, 9.6)	2 (0.6, 6.1)	$<$ 0.001	0.430
AFP, median (IQR)	2.57 (1.86, 3.45)	2.72 (2.07, 3.48)	0.091	0.085
CEA, median (IQR)	4.55 (2.14, 15.88)	3.55 (1.87, 14.01)	0.005	0.161
CA125, median (IQR)	10.6 (7, 19.05)	9.3 (6.2, 15.3)	$<$ 0.001	0.096
CA199, median (IQR)	18.5 (8.2, 46.25)	14.6 (7.2, 45.4)	0.036	0.085

Abbreviations: mGPS, modified Glasgow Prognostic Score; IQR, interquartile range; SMD, standardised mean difference; WBC, white blood cell count; RBC, red blood cell count; MCV, mean corpuscular volume; MCHC, mean corpuscular haemoglobin concentration; MCH, mean corpuscular haemoglobin; RDW-CV, red cell distribution width-coefficient of variation; Lymph, lymphocyte count; Mono, monocyte count; Neut, neutrophil count; Hct, haematocrit; Eos, eosinophil count; Baso, basophil count; Hb, haemoglobin; PLT, platelet count; MPV, mean platelet volume; PDW, platelet distribution width; ALT, alanine aminotransferase; AST, aspartate aminotransferase; GGT, gamma-glutamyl transferase; TBIL, total bilirubin; DBIL, direct bilirubin; ALB, albumin; GLO, globulin; TP, total protein; PA, prealbumin; TBA, total bile acid; Crea, creatinine; UA, uric acid; TC, total cholesterol; TG, triglyceride; HDL-C, high-density lipoprotein cholesterol; LDL-C, low-density lipoprotein cholesterol; Lpa, lipoprotein(a); apoAI, apolipoprotein AI; apoB, apolipoprotein B; K^+, potassium; Na^+, sodium; Cl^-, chloride; Ca, serum calcium; P, phosphorus; Mg^{2+}, magnesium; Glu, glucose; LDH, lactate dehydrogenase; ALP, alkaline phosphatase; ADA, adenosine deaminase; ChE, cholinesterase; Cys-C, cystatin C; FFA, free fatty acid; RBP, retinol-binding protein; CO_2CP, carbon dioxide combining power; eGFR, estimated glomerular filtration rate; Osm, osmolality; AFU, alpha-L-fucosidase; AG, anion gap; PT-INR, prothrombin time-international normalized ratio; PT, prothrombin time; TT, thrombin time; ATIII:A, antithrombin III activity; APTT, activated partial thromboplastin time; Fbg, fibrinogen; FDP, fibrin degradation products; CRP, C-reactive protein; AFP, alpha-fetoprotein; CEA, carcinoembryonic antigen; CA125, carbohydrate antigen 125; CA199, carbohydrate antigen 19-9. Continuous variables are presented as median (IQR), and categorical variables are presented as n (%). P values were calculated using the Mann–Whitney U test for continuous variables and Pearson's chi-square test, Yates' continuity correction or Fisher's exact test for categorical variables, as appropriate. SMD values are presented as absolute standardised differences, and an absolute SMD greater than 0.10 was considered indicative of a meaningful between-group difference.

Table S2: Hyperparameter optimisation results for the evaluated machine learning models and ensemble frameworks.

Model	Optimised Hyperparameters / Ensemble Weights	Five-fold CV AUC
**ANN**	hidden_layer_sizes: (50, 50)<br>activation: tanh<br>solver: adam<br>alpha: 0.0003<br>learning_rate_init: 0.0002	0.7244
**CatBoost**	iterations: 600<br>depth: 5<br>learning_rate: 0.0104<br>l2_leaf_reg: 5.8736<br>border_count: 129	0.7470
**Decision Tree**	max_depth: 14<br>min_samples_split: 31<br>min_samples_leaf: 5<br>criterion: gini	0.7123
**LightGBM**	n_estimators: 150<br>learning_rate: 0.0191<br>num_leaves: 141<br>max_depth: 11<br>subsample: 0.5597<br>colsample_bytree: 0.5284<br>reg_alpha: 0.8950<br>reg_lambda: 1.9029	0.7389
**Random Forest**	n_estimators: 500<br>max_depth: 13<br>min_samples_split: 18<br>min_samples_leaf: 4<br>max_features: sqrt	0.7438
**SVM**	C: 0.0073<br>gamma: scale<br>kernel: linear	0.7243

Continued on next page

Model	Optimised Hyperparameters / Ensemble Weights	Five-fold CV AUC
**XGBoost**	n_estimators: 250<br>max_depth: 4<br>min_child_weight: 2<br>learning_rate: 0.0176<br>subsample: 0.8149<br>colsample_bytree: 0.6264<br>gamma: 0.0002<br>reg_alpha: 0.0005<br>reg_lambda: 1.2965	0.7431
**TabICLv2**	Pre-trained tabular foundation model; zero-shot in-context learning; no task-specific hyperparameter tuning was performed	N/A
**Stacking**<br>*(SVM + CatBoost)*	svm_c: 1.9237<br>svm_gamma: auto<br>cb_iterations: 200<br>cb_depth: 8<br>cb_lr: 0.0091	0.7265
**Stacking**<br>*(TabICLv2 + XGBoost)*	TabICLv2: zero-shot in-context learning<br>xgb_estimators: 150<br>xgb_depth: 6<br>xgb_lr: 0.0965	0.7279
**SuperLearner-W**<br>*(NNLS)*	TabICLv2: 0.7792 (77.9%)<br>LR: 0.1276 (12.8%)<br>XGBoost: 0.0932 (9.3%)<br>CatBoost: 0.0000 (0.0%)<br>SVM: 0.0000 (0.0%)	N/A
**SuperLearner-SR**<br>*(L2 Ridge)*	TabICLv2: 0.7774 (77.7%)<br>XGBoost: 0.1246 (12.5%)<br>LR: 0.0980 (9.8%)<br>CatBoost: 0.0000 (0.0%)<br>SVM: 0.0000 (0.0%)	N/A

Abbreviations: CV, cross-validation; AUC, area under the receiver operating characteristic curve; ANN, artificial neural network; SVM, support vector machine; LR, logistic regression; NNLS, non-negative least squares.

Table S3: Performance comparison of machine learning models in the internal validation cohort using training-derived operating thresholds.

**Model**	**Threshold**	**AUC (95% CI)**	**Accuracy**	**Precision (PPV)**	**Sensitivity**	**Specificity**	**F1 score**	**Cohen's κ**	**NPV**
ANN	0.478	0.717 (0.662–0.771)	0.699	0.655	0.612	0.763	0.633	0.378	0.728
CatBoost	0.548	0.746 (0.693–0.799)	0.710	0.714	0.526	0.845	0.606	0.385	0.709
Decision Tree	0.552	0.673 (0.617–0.730)	0.638	0.566	0.618	0.652	0.591	0.267	0.699
LightGBM	0.484	0.720 (0.666–0.776)	0.694	0.652	0.592	0.768	0.621	0.365	0.719
Logistic Regression	0.522	0.717 (0.662–0.774)	0.705	0.698	0.533	0.831	0.604	0.376	0.708
Random Forest	0.361	0.737 (0.686–0.791)	0.652	0.562	0.809	0.536	0.663	0.326	0.793
SVM	0.492	0.717 (0.662–0.774)	0.696	0.659	0.586	0.778	0.620	0.369	0.719
Stacking (SVM + CatBoost)	0.492	0.709 (0.652–0.764)	0.688	0.649	0.572	0.773	0.608	0.351	0.711
Stacking (TabICLv2 + XGB)	0.550	0.726 (0.673–0.781)	0.721	0.777	0.480	0.899	0.593	0.399	0.702
SuperLearner-SR	0.466	0.751 (0.700–0.803)	0.705	0.653	0.645	0.749	0.649	0.394	0.742
SuperLearner-W	0.486	0.752 (0.702–0.803)	0.716	0.679	0.625	0.783	0.651	0.412	0.740
TabICLv2	0.525	0.753 (0.701–0.804)	0.721	0.710	0.579	0.826	0.638	0.415	0.728
XGBoost	0.467	0.742 (0.689–0.796)	0.719	0.676	0.645	0.773	0.660	0.420	0.748

Abbreviations: ANN, artificial neural network; AUC, area under the receiver operating characteristic curve; CI, confidence interval; NPV, negative predictive value; PPV, positive predictive value; SVM, support vector machine. Model-specific operating thresholds were determined from out-of-fold predictions in the training cohort by maximising the Youden index and were subsequently applied unchanged to the internal validation cohort. All reported performance metrics were calculated in the internal validation cohort.

SUPPLEMENTARY FIGURES

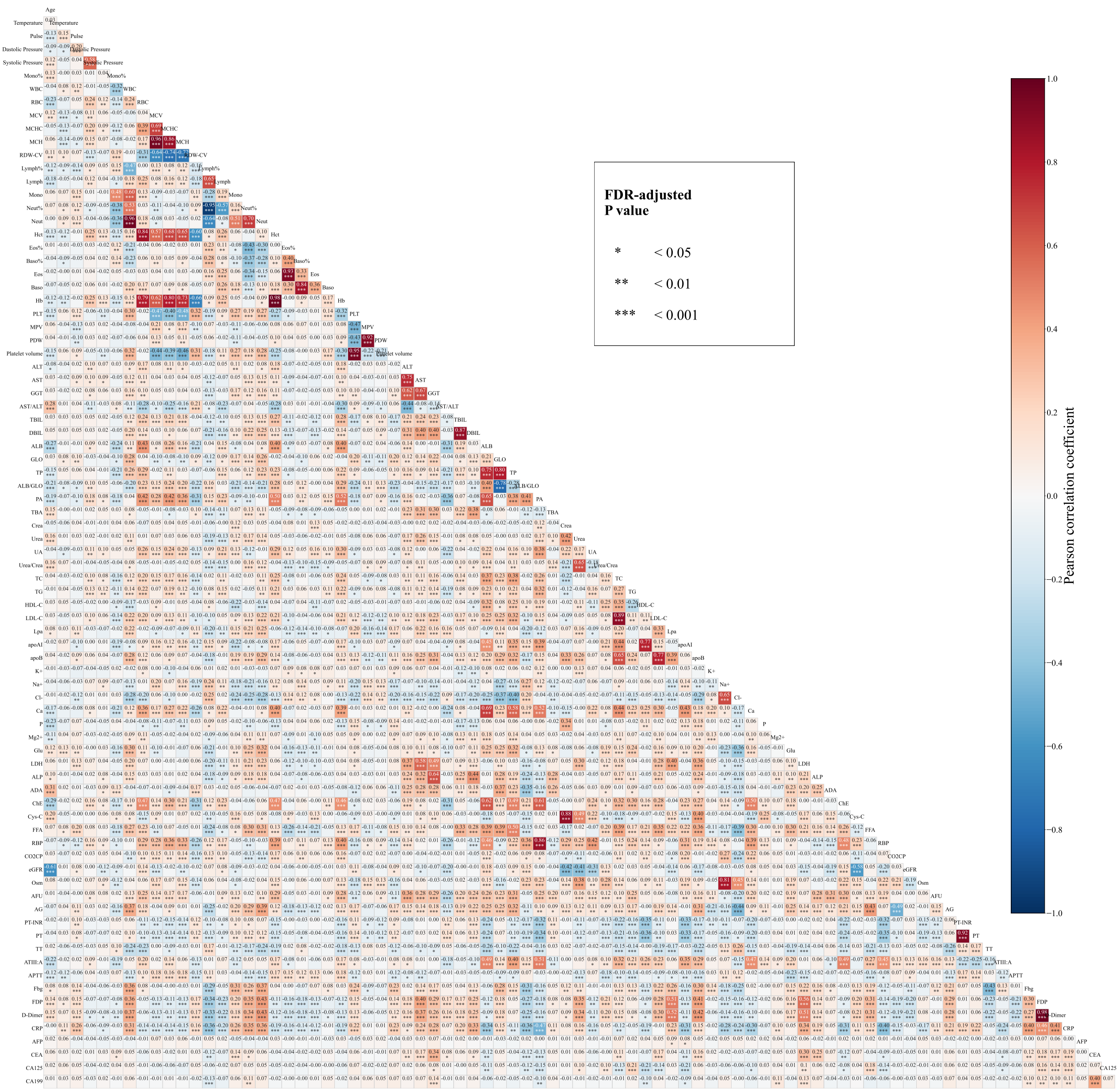

Supplementary Figure S1. Correlation heatmap of continuous baseline variables. Values represent Pearson correlation coefficients. Statistical significance based on Benjamini–Hochberg false discovery rate-adjusted P values was indicated as follows: * $P < 0.05$, ** $P < 0.01$, and *** $P < 0.001$.

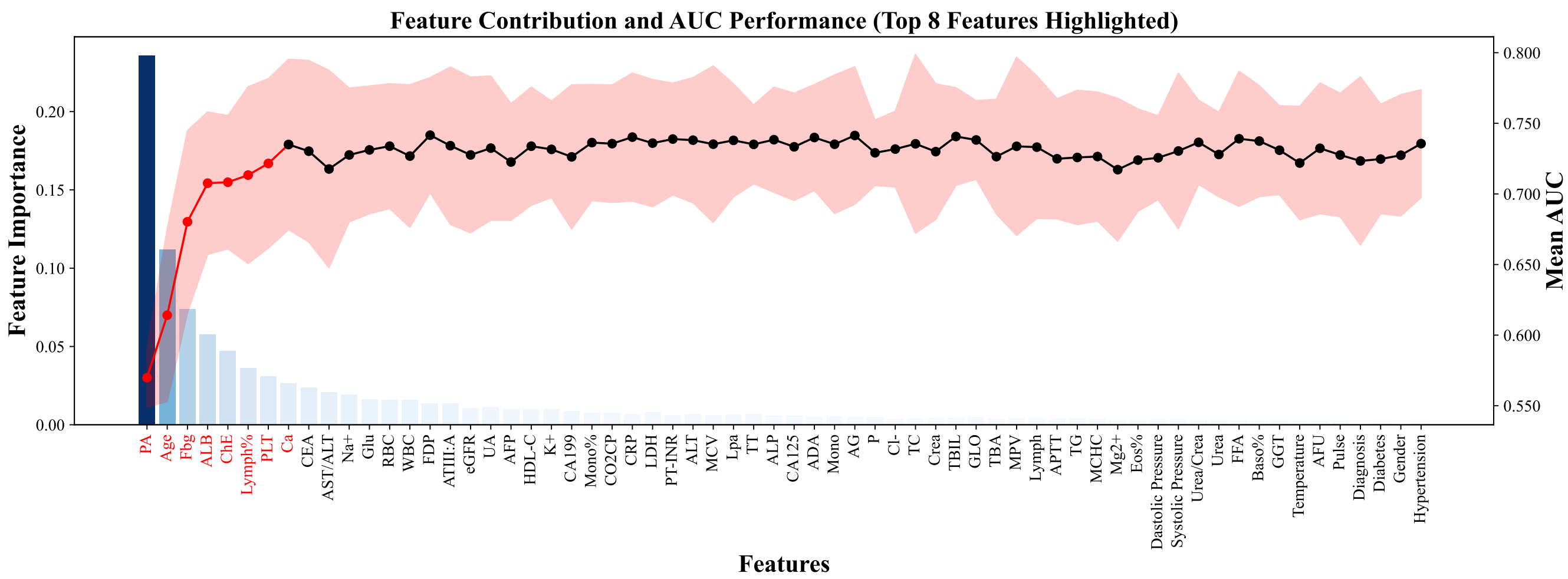

Supplementary Figure S2. Recursive feature elimination curve for core predictor selection. RFE was performed using a random forest classifier with five-fold cross-validation. Blue bars indicate the normalised feature importance of candidate predictors. The line plot shows the mean area under the receiver operating characteristic curve after sequential feature inclusion, and the shaded area represents the 95% confidence interval of the cross-validation AUC estimates. The eight predictors highlighted in red were retained as the final core feature subset for model development. RFE, recursive feature elimination; AUC, area under the curve.

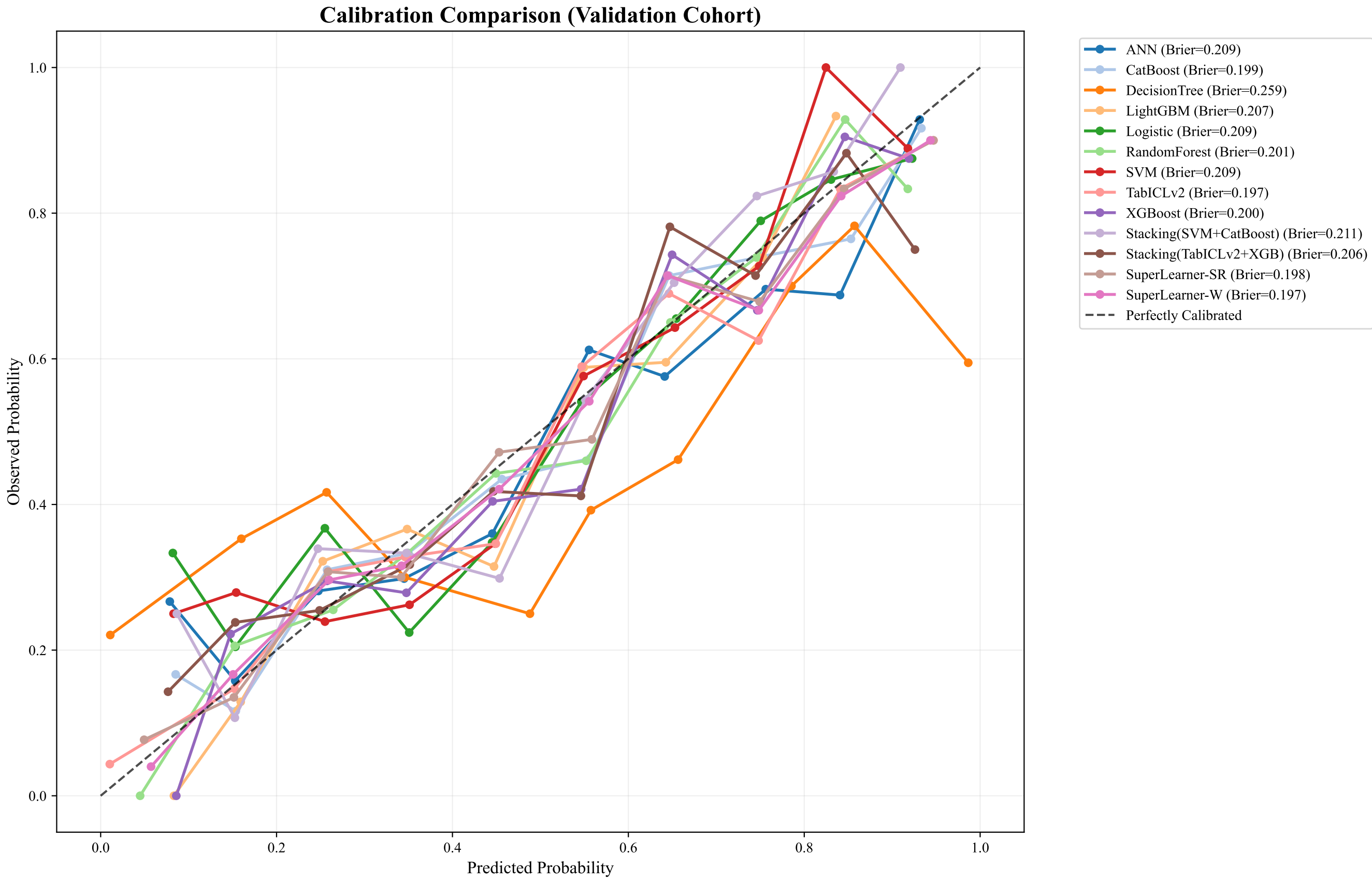

Supplementary Figure S3. Calibration curves of the evaluated predictive models in the internal validation cohort. The x-axis represents the predicted probability of postoperative mGPS = 2, and the y-axis represents the observed probability. The dashed diagonal line indicates perfect calibration. Brier scores are shown in the legend, with lower values indicating better probabilistic calibration. mGPS, modified Glasgow Prognostic Score.

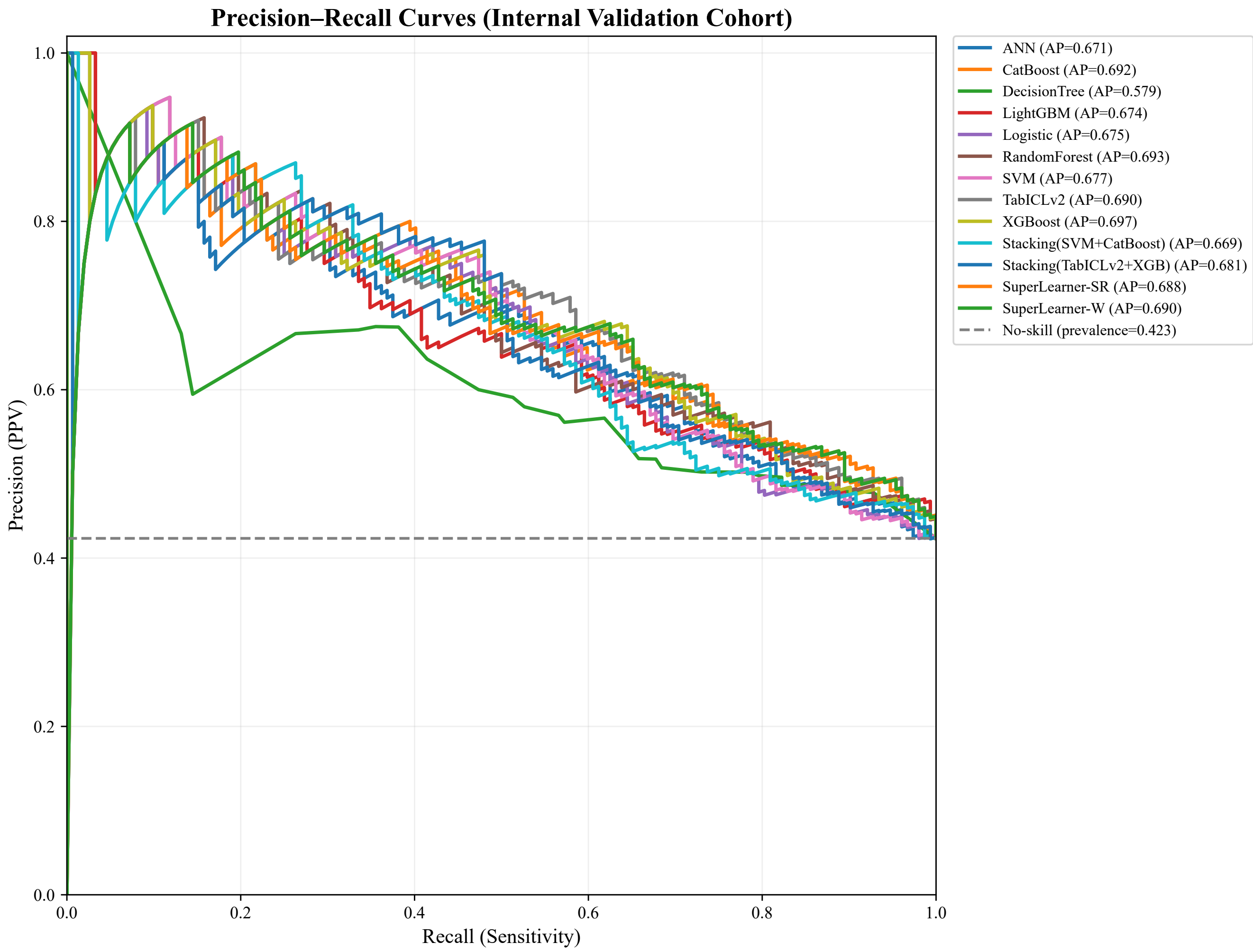

Supplementary Figure S4. Precision–recall curves of the evaluated predictive models in the internal validation cohort. Curves were constructed using continuous predicted probabilities without applying a fixed classification threshold. Average precision (AP) is reported for each model in the figure legend. The horizontal dashed line represents the no-skill reference corresponding to the observed prevalence of postoperative mGPS = 2 in the internal validation cohort (152/359, 42.3%). PR, precision–recall; AP, average precision; mGPS, modified Glasgow Prognostic Score.

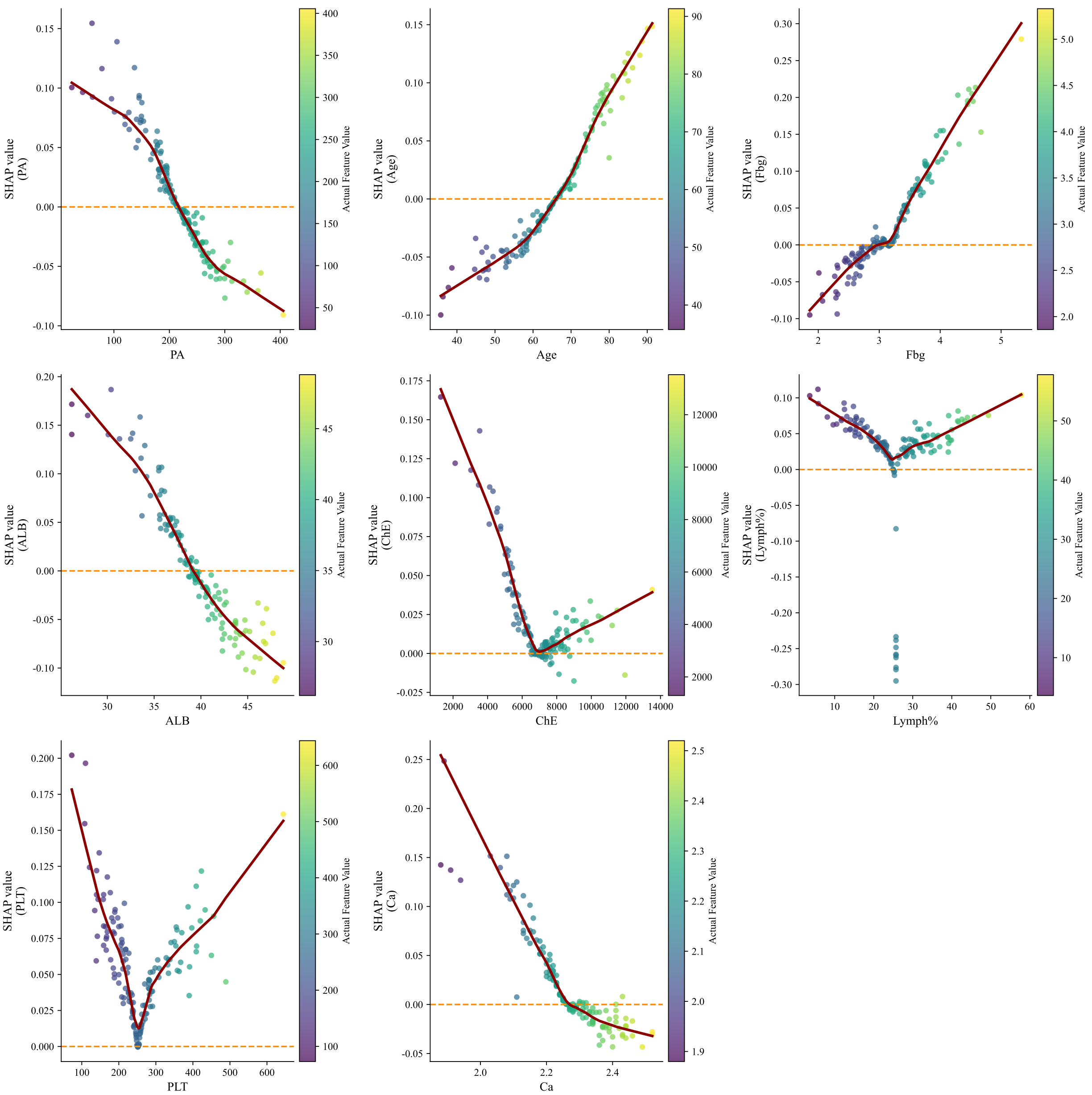

Supplementary Figure S5. SHAP dependence plots for the eight core predictive features of the TabICLv2 model. Each point represents one of the 150 interpreted patients in the internal validation cohort. The x-axis indicates the observed value of each predictor, and the y-axis indicates its SHAP value, reflecting the direction and magnitude of the feature contribution to the model output towards postoperative mGPS = 2. The red curves represent LOWESS-smoothed trends, and the orange dashed horizontal lines indicate a SHAP value of zero. SHAP, SHapley Additive exPlanations; mGPS, modified Glasgow Prognostic Score; PA, prealbumin; Fbg, fibrinogen; ALB, albumin; ChE, cholinesterase; Lymph%, lymphocyte percentage; PLT, platelet count; Ca, serum calcium.

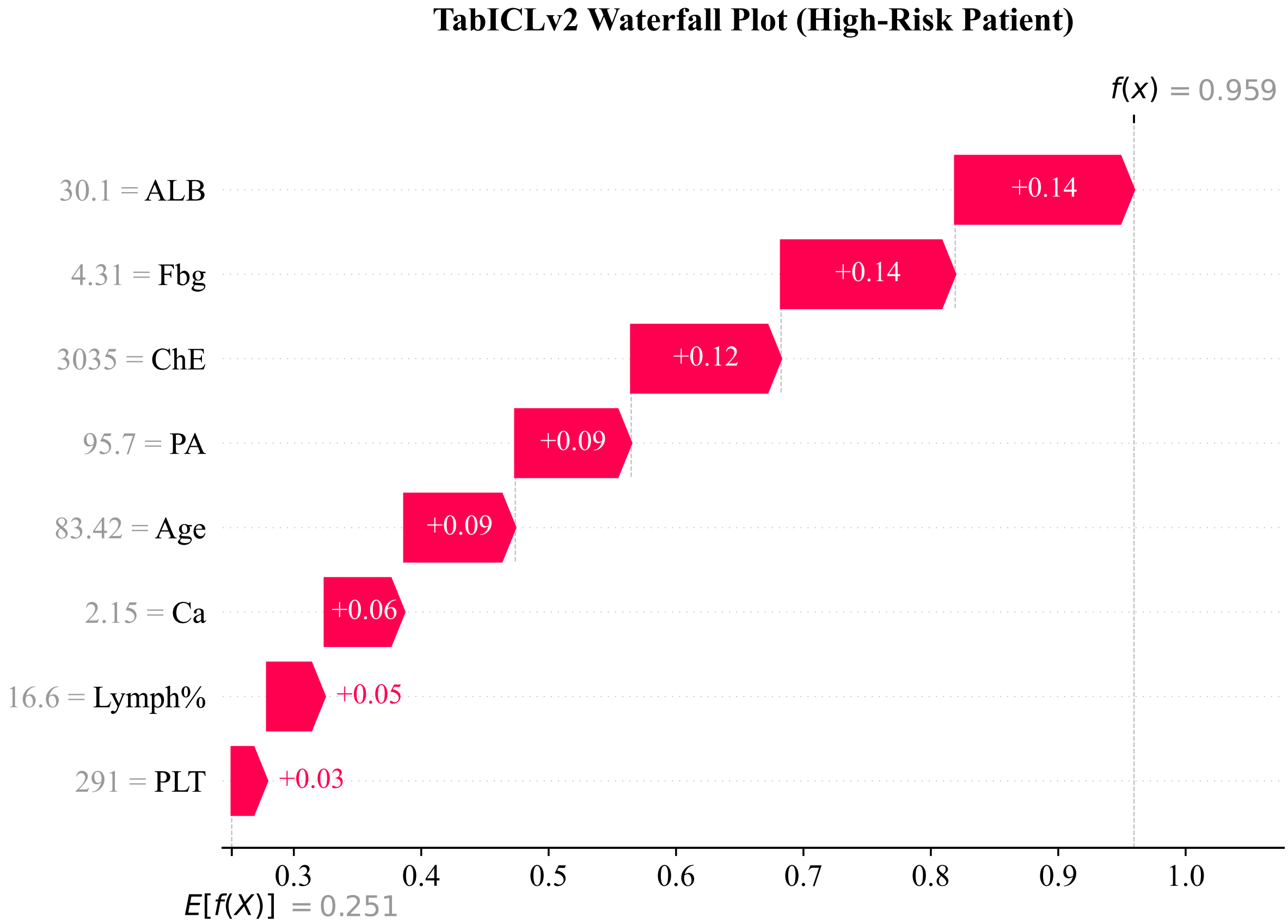

Supplementary Figure S6A. Patient-level SHAP waterfall plot for the illustrative high-risk patient. The plot decomposes the explained model output from the reference value $E[f(X)]$ to the patient-specific output $f(x)$. Positive SHAP values shift the model output towards postoperative mGPS = 2, whereas negative SHAP values shift it away from postoperative mGPS = 2. The displayed $E[f(X)]$ and $f(x)$ values are on the SHAP explainer's output scale and should not be interpreted as calibrated probabilities. SHAP, SHapley Additive exPlanations; mGPS, modified Glasgow Prognostic Score.

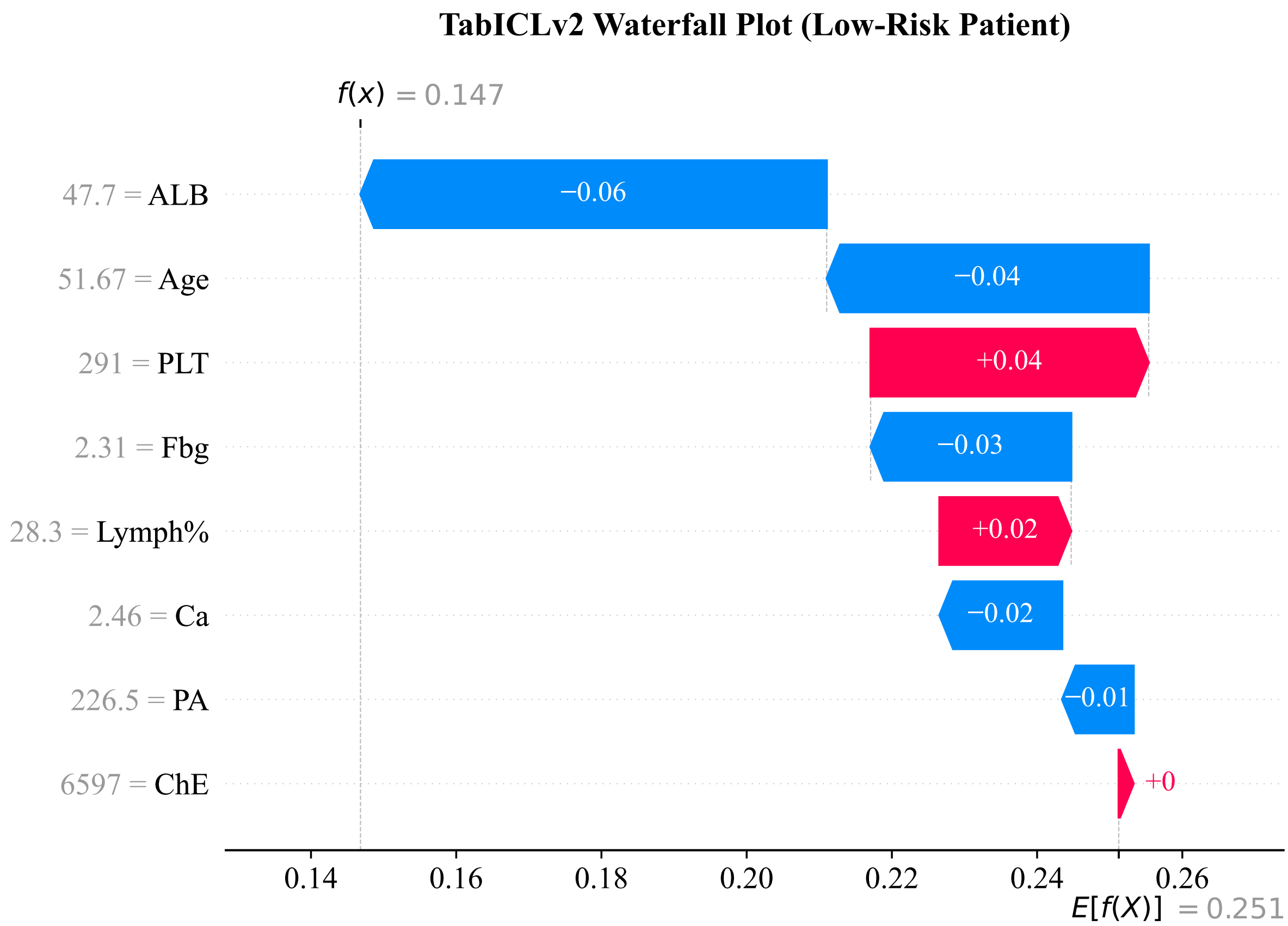

Supplementary Figure S6B. Patient-level SHAP waterfall plot for the illustrative low-risk patient. The plot decomposes the explained model output from the reference value $E[f(X)]$ to the patient-specific output $f(x)$. Positive SHAP values shift the model output towards postoperative mGPS = 2, whereas negative SHAP values shift it away from postoperative mGPS = 2. The displayed $E[f(X)]$ and $f(x)$ values are on the SHAP explainer's output scale and should not be interpreted as calibrated probabilities. SHAP, SHapley Additive exPlanations; mGPS, modified Glasgow Prognostic Score.

(A)

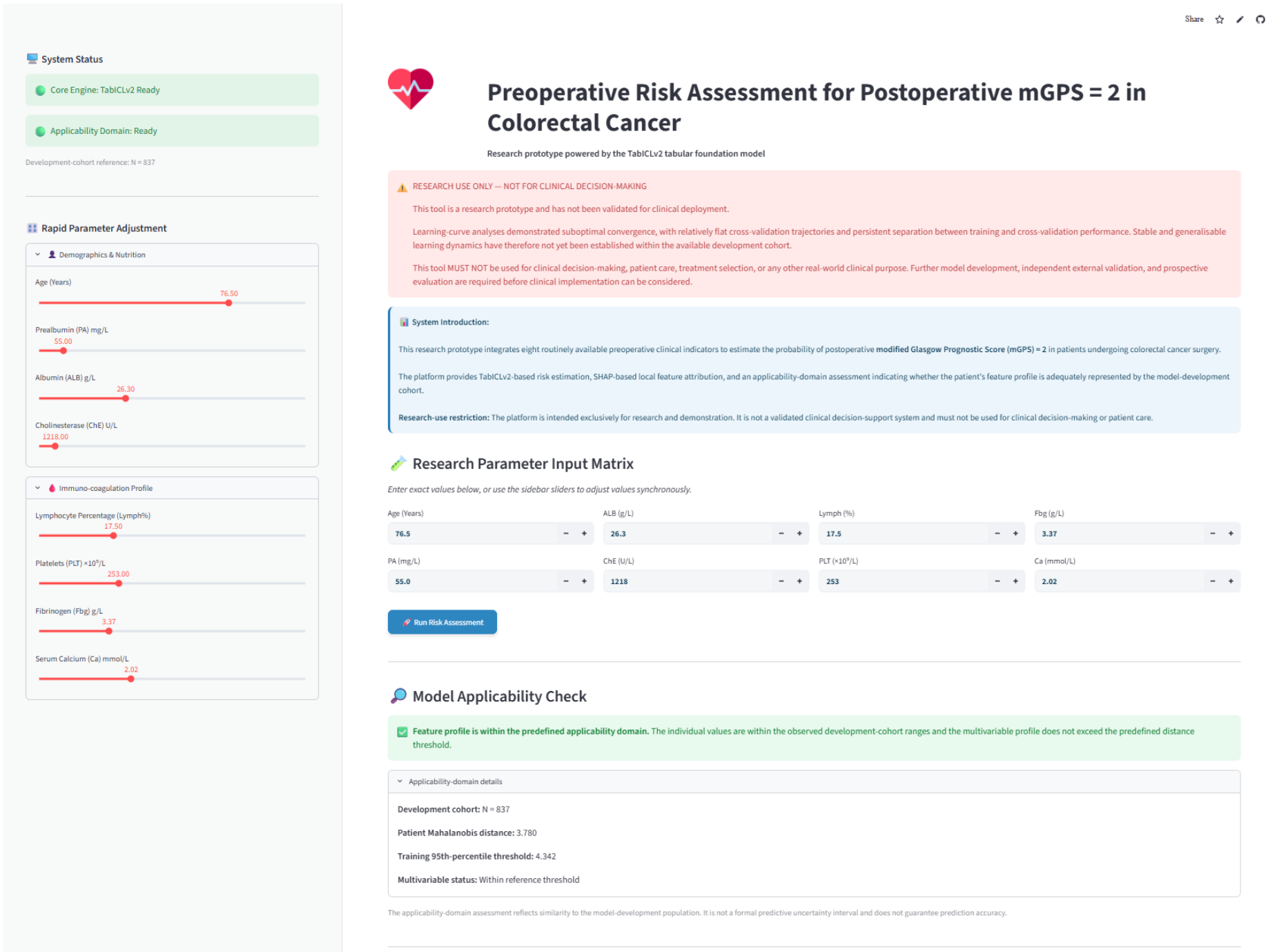

(B)

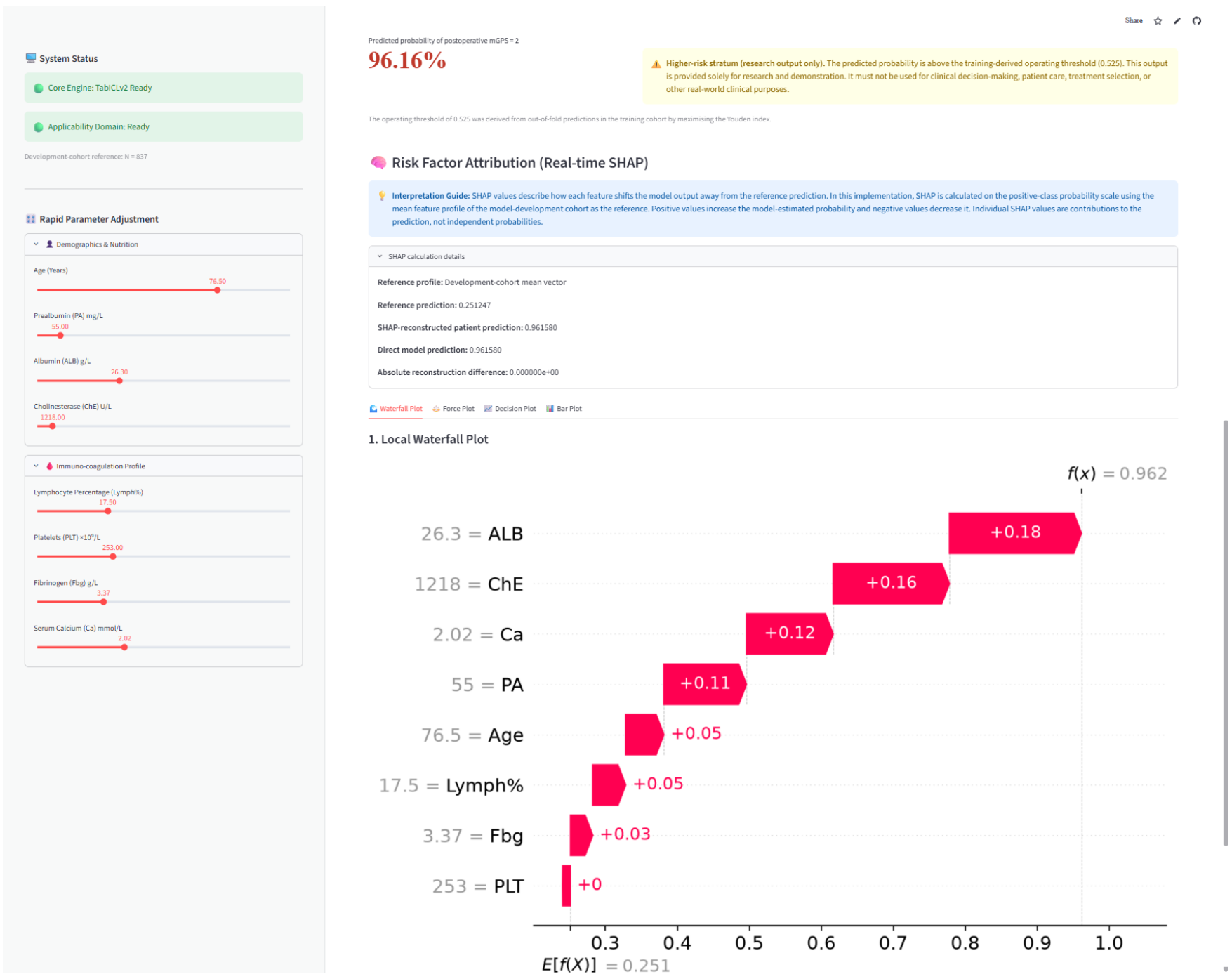

Supplementary Figure S7. Representative screenshots of the web-based research prototype for individualised prediction of postoperative mGPS = 2. **(A)** Upper interface showing the prominent research-use warning, system introduction and patient input section. **(B)** Model-output interface showing the predicted probability, applicability-domain assessment and real-time SHAP-based local feature attribution. The platform explicitly states that it is intended exclusively for research and demonstration and must not be used for clinical decision-making or patient care. SHAP, SHapley Additive exPlanations; mGPS, modified Glasgow Prognostic Score.

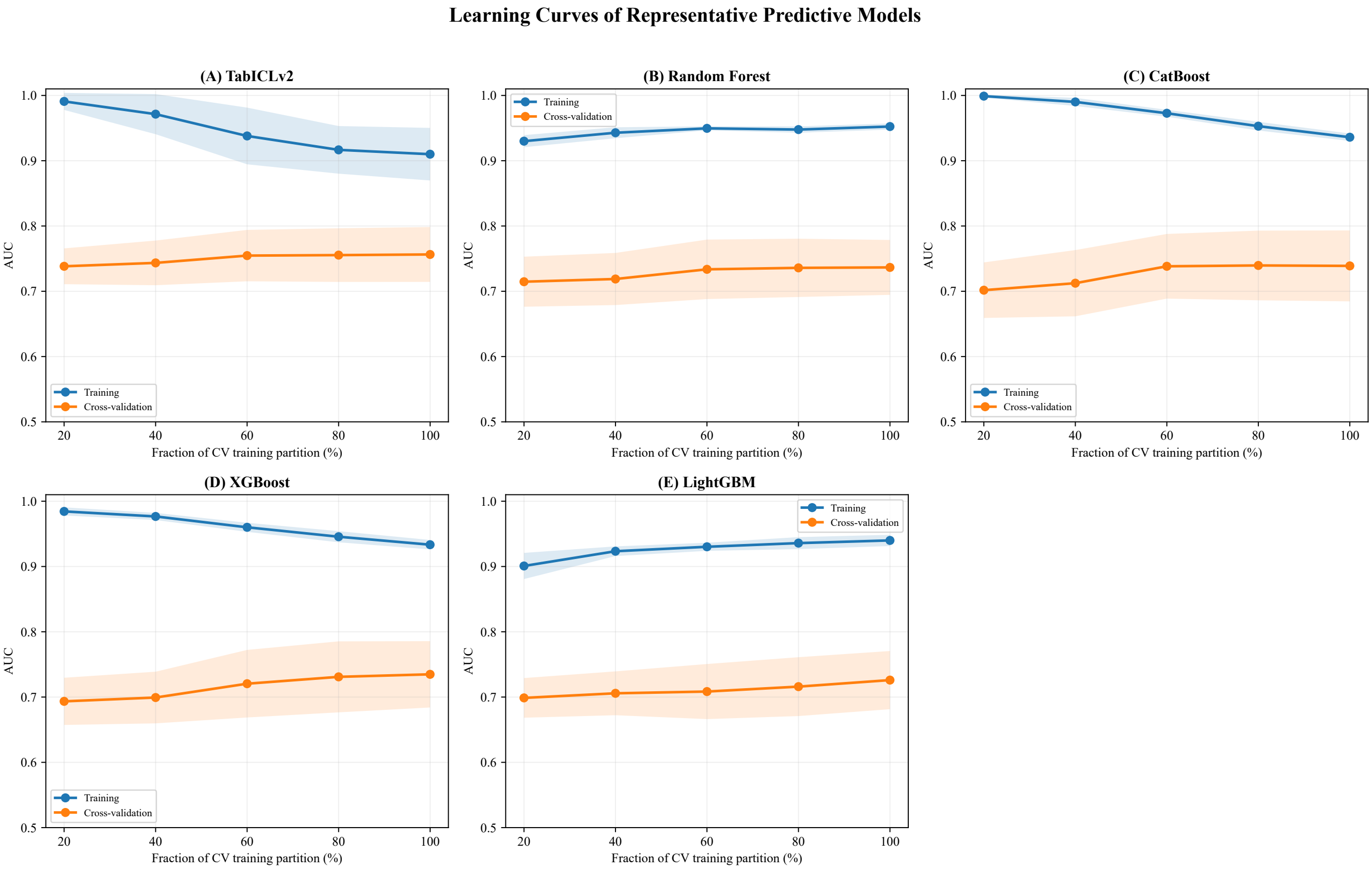

Supplementary Figure S8. Learning curves of representative predictive models evaluated within the original training cohort. **(A)** TabICLv2; **(B)** Random Forest; **(C)** CatBoost; **(D)** XGBoost; and **(E)** LightGBM. Stratified five-fold cross-validation was used, and 20%, 40%, 60%, 80% and 100% of each fold-specific training partition were used for model fitting. Lines indicate the mean training and cross-validation AUC across five folds, and shaded regions represent ± 1 standard deviation. Previously selected model specifications were retained without additional hyperparameter optimisation. The independent internal validation cohort was not used in this analysis. Across the evaluated models, cross-validation trajectories showed only modest changes with increasing training-set size, while persistent separation between training and cross-validation performance indicated suboptimal convergence within the available cohort. AUC, area under the receiver operating characteristic curve.
